# A miniaturized dual-port circularly polarized MIMO patch antenna for SAR-compliant wearable X-band communication systems

**DOI:** 10.1038/s41598-026-47610-7

**Published:** 2026-04-09

**Authors:** Josephine Pon Gloria, Muthu Manickam Anbarasu, Javid Ali Liakath, D. Siva Sundhara Raja, A. Priya, Immanuel Prabaharan S, D. Rajesh Kumar, Om Prakash Kumar

**Affiliations:** 1 Department of Electronics and Communication Engineering, Vel Tech Rangarajan Dr. Sagunthala R&D Institute of Science and Technology, Chennai, 600062 India; 2 Department of Electronics and Communication Engineering, Shanmuganathan Engineering College, Pudukottai, 622507 India; 3 Department of CSE (Cyber Security), St. Joseph’s Institute of Technology, Chennai, 600119 India; 4 Department of Electronics and Communication Engineering, SACS MAVMM Engineering College, Madurai, 625301 India; 5 Department of Electronics and Communication Engineering, B.S. Abdur Rahman Crescent Institute of Science and Technology, Vandalur, Chennai, 600048 India; 6 Department of Electrical and Electronics Engineering, Academy of Maritime Education and Training (AMET) Deemed to be University, Tamil Nadu, Chennai, 603 112 India; 7 Manipal Institute of Technology, Manipal Academy of Higher Education, Manipal, 576104 India

**Keywords:** Circularly polarized MIMO antenna, X-band wearable antenna, Specific absorption rate (SAR), Envelope correlation coefficient (ECC), Circular microstrip patch, Front-to-back ratio (FBR), Body-centric communication, Port isolation, SDG-9 (Industry, Innovation and Infrastructure), SDG-11 (Sustainable Cities and Communities) and SDG-3 (Good Health and Well-Being), Engineering, Materials science, Physics

## Abstract

Wearable communication systems operating at X-band demand antenna designs that simultaneously satisfy stringent miniaturization requirements, maintain stable circular polarization, support MIMO diversity, and ensure electromagnetic safety for body-worn deployment. Existing designs in the literature largely address these challenges in isolation, with most reported X-band circularly polarized antennas remaining physically large and lacking rigorous wearable safety validation. This paper presents a compact dual-port circularly polarized MIMO antenna specifically engineered for wearable X-band applications in the 9.0–9.3 GHz range. The principal novelty lies in the simultaneous achievement of ultra-compact form factor, stable circular polarization, effective port isolation, and full SAR compliance within a single passive, single-layer architecture — a combination absent in prior art. Circular polarization is realized through geometry-driven feed offset and patch curvature without external matching networks or multi-feed structures, significantly simplifying the design. The antenna demonstrates strong MIMO diversity performance with low envelope correlation, confirming spatially uncorrelated radiation despite compact inter-element spacing. On-body SAR evaluation using a realistic hand model confirms compliance with international safety standards, validating the design for prolonged wearable use. Simulated results are experimentally verified, with close agreement across all key performance metrics. The proposed antenna advances the state of the art by delivering a practical, application-driven solution that integrates miniaturization, polarization stability, MIMO capability, and wearable safety — making it suitable for next-generation body-centric X-band communication platforms.

## Introduction

The X-band, defined by IEEE Standard 521–2002 as spanning 8.0–12.0 GHz, encompasses several ITU-designated primary allocations including 9.0–9.2 GHz for earth exploration satellite services, 9.2–9.5 GHz for maritime navigation radar, and 9.3–9.5 GHz for meteorological and airborne radar systems^1,2^. Within this band, the 9.0–9.3 GHz sub-region is of particular interest for compact short-range wireless platforms because it combines a free-space wavelength of approximately 33 mm — small enough to enable patch radiators with sub-centimeter dimensions — with propagation characteristics that are superior to millimeter-wave bands (above 30 GHz), where atmospheric oxygen absorption exceeds 10 dB/km and rain attenuation reaches 0.01 dB/km at 9 GHz compared to over 1 dB/km at 60 GHz^3,4^. Unlike the sub-6 GHz spectrum, which is densely occupied by IEEE 802.11b/g/n/ax Wi-Fi at 2.4 and 5 GHz, Bluetooth Low Energy at 2.4 GHz, and 5G NR FR1 bands from 600 MHz to 5.925 GHz, the 9.0–9.3 GHz range carries significantly lower commercial device density, offering reduced co-channel interference for dedicated wearable sensing and communication links^5,6^. These spectrum characteristics — combined with the achievable antenna miniaturization at 9 GHz — establish a technically grounded basis for targeting this specific sub-band for compact body-worn applications, rather than operating at lower bands where spectral congestion limits performance or at millimeter-wave bands where propagation losses demand impractically high transmit power for body-area links^7,8^.

Wearable and body-centric platforms operating at 9.0–9.3 GHz face five simultaneous and interdependent antenna design constraints that collectively define the challenge addressed in this work. First, the physical footprint of the antenna must not exceed the surface area available on body-worn devices; existing X-band CP designs in literature report dimensions ranging from 60 × 60 mm^2^ to 140 × 140 mm^2^^6–8^, which are incompatible with wrist-worn or patch-type wearable nodes where available antenna area is typically below 50 × 20 mm^2^. Second, body proximity at 9 GHz introduces permittivity loading from human tissue (relative permittivity εr ≈ 7–11 and conductivity σ ≈ 1.3–1.7 S/m at 9 GHz for skin tissue), which causes resonance frequency shifts exceeding 200 MHz and gain reductions of 2–4 dB if the antenna ground plane does not adequately shield the radiating element from the body^9,10^. Third, the FCC (CFR 47 Part 2.1093) and ICNIRP 1998 guidelines impose mandatory SAR limits of 1.6 W/kg averaged over 1 g of tissue and 2.0 W/kg averaged over 10 g of tissue, respectively, making electromagnetic exposure analysis an obligatory validation step for any antenna intended for prolonged contact with the human body^11,12^. Fourth, MIMO operation at X-band requires inter-port isolation better than − 15 dB and an envelope correlation coefficient (ECC) below 0.5 — and ideally below 0.1 — to ensure that the two antenna ports sample sufficiently uncorrelated channel realizations, which is particularly demanding when compact spacing forces elements to within half a wavelength of each other (λ/2 ≈ 16.7 mm at 9 GHz)^13–15^. Fifth, circular polarization — defined by a 3 dB axial ratio bandwidth — is required to provide immunity against the polarization mismatch that arises from the continuous and unpredictable rotation of a wrist-worn or body-worn antenna during movement, where orientation changes of 90° or more can produce up to 20 dB of signal loss in a linearly polarized link^16–18^. No published X-band design has simultaneously satisfied all five of these constraints within a single compact passive architecture.

Early research on compact circularly polarized patch antennas established the perturbation technique as the primary method for generating CP from a single feed. Chen et al.^17^ achieved CP operation at 2.45 GHz using a square-ring patch with an embedded cross-strip, reporting a 3 dB axial ratio bandwidth of 1.2% and a realized gain of 3.5 dBi from a 40 × 40 mm^2^ element. Orr et al.^18^ demonstrated a Fabry–Pérot cavity-based CP antenna with a 10.3% impedance bandwidth and 12.5 dBi broadside gain, but with an antenna height of 28 mm — nearly one wavelength at the operating frequency — making it unsuitable for planar wearable integration. Stacked-patch hybrid perturbation designs in^19^ extended the 3 dB axial ratio bandwidth to 5.2% but required two substrate layers and a total profile of 6.4 mm, and the resulting structure occupied a footprint of 70 × 70 mm^2^. Higher-order mode suppression techniques reported in^7^ produced dual-frequency CP operation at S- and X-bands from a single element, but the antenna required a 85 × 85 mm^2^ footprint with an axial ratio bandwidth of only 0.9% at the X-band resonance. In all these perturbation-based designs, the sensitivity of the phase balance to dimensional tolerances is a critical limitation: a 0.1 mm fabrication error in the perturbation stub length causes a phase imbalance exceeding 5° at 9 GHz, which increases the axial ratio from below 1 dB to above 3 dB and degrades polarization purity^7,17^. This sensitivity is further amplified in dual-port MIMO configurations where inter-element coupling perturbs the orthogonal mode excitation required for stable CP.

CubeSat and nanosatellite programs drove significant advances in compact X-band antenna design from 2014 onward, but exclusively for space-platform deployment. Deployable Ka-band reflectarray antennas demonstrated in^20,21^ achieved gains exceeding 20 dBi within stowed volumes fitting a 3U CubeSat (100 × 100 × 340 mm^3^) using shape-memory polymer hinges for deployment actuation. Warren et al.^22^ reported a deployable S-band antenna for 6U CubeSats achieving 18.5 dBi gain with a 340 MHz impedance bandwidth, but requiring a deployment mechanism occupying the full 200 × 200 mm^2^ aperture. V-band inter-CubeSat link antennas in^3^ demonstrated 22 dBi gain and 2.4° half-power beamwidth at 60 GHz using a bull’s-eye cavity structure of 80 × 80 mm^2^. Surveys in^4,23^ identified the 9.3–9.5 GHz sub-band as the ITU-allocated primary window for SAR (synthetic aperture radar) payloads on small satellites, with minimum required antenna gains of 15 dBi for 1 m ground resolution at 500 km orbital altitude. Without exception, all these designs employ deployable structures, multi-layer configurations, or thermally actuated elements whose mechanical complexity is incompatible with the planar, permanently conformal, and flexible construction required for body-worn antennas.

Shared-aperture architectures were explored to combine X-band CP operation with multi-band or MIMO functionality within compact structures. Wang et al.^24^ proposed a shared-aperture CP antenna using metallic isolation columns between L-band and S-band sub-apertures, achieving isolation of 22 dB between bands but requiring a total footprint of 75 × 75 mm^2^ and a fabrication process involving precision-milled metal posts of 3 mm diameter. Xie et al.^25^ reported an L/S dual-band shared-aperture CP structure with measured 3 dB axial ratio bandwidths of 4.1% at 1.57 GHz and 3.8% at 2.45 GHz from a 65 × 65 mm^2^ substrate, but without any MIMO port configuration or isolation analysis between radiating elements. For X-band airborne synthetic aperture radar, Bandi et al.^6^ introduced a single-layer S/X-band shared-aperture design with four MIMO ports at X-band, achieving inter-port isolation of − 18 dB and a realized gain of 8.2 dBi at 9.5 GHz from an 82 × 82 mm^2^ substrate — however, no SAR (specific absorption rate) analysis was conducted, no on-body testing was performed, and the 82 × 82 mm^2^ footprint far exceeds the dimensional limit for wearable deployment^6,24^.

Wearable-specific MIMO antenna research has concentrated predominantly on sub-6 GHz bands, with very limited work at X-band. Sid et al.^9^ demonstrated a flexible dual-band wearable antenna on a bio-based substrate operating at 2.4 and 5.8 GHz, reporting a SAR value of 0.87 W/kg (1 g tissue) and a peak gain of 3.2 dBi, but using linear polarization and without MIMO capability. Manepalli^10^ reported a wideband circularly polarized textile antenna at 2.45 GHz with 4.1 dBi gain on a 90 × 70 mm^2^ jeans fabric substrate, but as a single-port element without port isolation or ECC characterization. A sixteen-port flexible CP UWB MIMO design in^13^ achieved ECC below 0.08 across 3.1–10.6 GHz and isolation better than − 15 dB, but required a footprint of 120 × 60 mm^2^ and a substrate thickness of 1.2 mm in a bent configuration — and critically, no SAR analysis was included. A mmWave CP MIMO design using characteristic mode theory at 28 GHz^14^ achieved isolation of − 18 dB and ECC below 0.08 from a 70 × 70 mm^2^ structure, but X-band operation and SAR compliance were not addressed. An implantable CP MIMO antenna in^26^ demonstrated compact 35 × 35 mm^2^ dimensions with SAR compliance on skin, brain, and heart phantoms, but the implantable nature of the design — operating inside biological tissue — precludes its use as a surface-worn wearable device. Metamaterial-loaded MIMO designs in^15^ achieved ECC below 0.04 and isolation of − 22 dB at sub-6 GHz from a 60 × 60 mm^2^ substrate, but without circular polarization and with no on-body analysis. Ultra-miniaturized MIMO designs in^27^ reported compact 45 × 45 mm^2^ dimensions with − 14 dB isolation at medical implant bands, but with ECC as high as 0.15 and no CP capability. A compact CP wearable antenna at 5.8 GHz in^28^ achieved an axial ratio bandwidth of 3.8% and gain of 5 dBi from a 65 × 65 mm^2^ structure, but as a single-port element without MIMO diversity analysis. A low-SAR wearable antenna using an FSS reflector in^29^ demonstrated SAR reduction from 2.8 W/kg to 0.73 W/kg (1 g tissue) at 2.4 GHz from an 85 × 50 mm^2^ footprint, but used linear polarization and had no MIMO configuration.

The foregoing review, quantitatively summarized in Table 3, identifies three specific and simultaneously unresolved gaps in the literature. First, no existing design achieves all of the following together within a single X-band structure: dual-port MIMO operation, stable circular polarization (AR < 3 dB), inter-port isolation better than − 12 dB, ECC below 0.05, and verified SAR compliance below the FCC/ICNIRP limits — designs that address three or four of these criteria consistently fail on the remainder. Second, all reported X-band CP designs occupy footprints from 60 × 60 mm^2^ to 140 × 140 mm^2^, while no X-band CP MIMO antenna with a footprint below 50 × 20 mm^2^ has been reported. Third, full MIMO diversity characterization — comprising ECC, diversity gain (DG), channel capacity loss (CCL), and mean effective gain (MEG) — has never been reported together for an X-band CP antenna with on-body SAR validation. The present work resolves all three gaps through a compact dual-port circularly polarized MIMO patch antenna measuring 40 × 15 × 1.6 mm^3^, operating over 9.0–9.3 GHz on an FR-4 substrate (εr = 4.4, tanδ = 0.02). Circular polarization is generated entirely through the geometry of the coaxial feed offset and circular patch curvature — without slits, truncated corners, or external matching networks — producing a 90° phase quadrature between orthogonal TM₁₁ modes through an asymmetric feed capacitance of Cr = 0.11 pF. The design achieves measured return loss below − 15 dB, isolation better than − 12 dB, axial ratio below 3 dB, ECC below 0.05, and SAR values of 0.96 W/kg (1 g) and 0.726 W/kg (10 g) — all within a footprint that is at least 2.25 × smaller than any comparable X-band CP design in the literature. The remainder of this paper is organized as follows: Section II describes the antenna geometry and equivalent circuit; Section III presents the parametric study; Sections IV and V discuss simulated and measured results; Section VI covers on-body SAR analysis; Section VII provides state-of-the-art comparison; and Section VIII concludes the paper.

## Antenna design and configuration

### Substrate and topology selection

The substrate material directly governs the resonant frequency, radiation efficiency, and physical compactness of the antenna. FR-4 (εr = 4.4, tanδ = 0.02, h = 1.6 mm) is selected as the substrate material because its relatively high permittivity reduces the physical patch dimensions by a factor of 1/√εr compared to free-space dimensions, enabling the compact 40 × 15 mm^2^ footprint required for wearable integration. While lower-loss substrates such as Rogers RT/duroid 5880 (tanδ = 0.0009) offer superior radiation efficiency, they are significantly more expensive and less suitable for low-cost wearable fabrication. At 9.1 GHz, the calculated radiation efficiency on FR-4 remains above 80%, which is acceptable for the target application.

A circular microstrip patch topology is specifically chosen over rectangular or square alternatives for two electromagnetic reasons. First, the circular patch supports the dominant TM₁₁ mode, which inherently produces two degenerate orthogonal field configurations of equal amplitude separated by 90° in the azimuthal plane — the fundamental precondition for circular polarization. In rectangular patches, achieving this orthogonality requires additional perturbation elements such as corner truncations or diagonal slots that increase structural complexity and sensitivity to fabrication tolerances. Second, the circular boundary ensures a symmetric surface current distribution that minimizes cross-polarization radiation and stabilizes the axial ratio across the operating bandwidth, which is critical for maintaining CP performance under the body-proximity detuning effects present in wearable scenarios.

### Patch radius determination

The physical radius R of the circular patch is the primary parameter governing the resonant frequency of the TM₁₁ mode. It is computed using the cavity model, which accounts for fringing field effects at the patch periphery through an effective radius correction. The governing expressions are:

1 $$R = \frac{F}{{\sqrt {1 + \frac{2h}{{\pi \varepsilon_{r} F}}\left[ {{\mathrm{ln}}\left( {\frac{\pi F}{{2h}}} \right) + 1.7726} \right]} }}$$

2 $$F = \frac{{8.791 \times 10^{9} }}{{f_{r} \sqrt {\varepsilon_{r} } }}$$

where fr is the target resonant frequency (Hz), h is the substrate thickness (cm), and εr is the substrate relative permittivity. The constant 1.7726 in Eq. (1) arises from the Euler–Mascheroni approximation used in the fringing field correction. Substituting fr = 9.1 GHz, εr = 4.4, and h = 0.16 cm into Eq. (2):

$$F = \frac{{8.791 \times 10^{9} }}{{9.1 \times 10^{9} \times \sqrt {4.4} }} = \frac{8.791}{{9.1 \times 2.098}} = 0.4603{\text{ cm}}$$

Substituting F = 0.4603 cm and h = 0.16 cm into Eq. (1):

$$R = \frac{0.4603}{{\sqrt {1 + \frac{2 \times 0.16}{{\pi \times 4.4 \times 0.4603}}\left[ {{\mathrm{ln}}\left( {\frac{\pi \times 0.4603}{{2 \times 0.16}}} \right) + 1.7726} \right]} }} \approx 0.60{\text{ cm}} = 6{\text{ mm}}$$

This analytically computed radius of R = 6 mm sets the initial design value, which is subsequently confirmed by full-wave simulation to produce the TM₁₁ resonance at 9.1 GHz with return loss below − 15 dB. The close agreement between the cavity model prediction and the simulated resonance validates the suitability of Eqs. (1) and (2) for this substrate–frequency combination. Any deviation from R = 6 mm — as demonstrated in the parametric study in Section III — shifts the resonance frequency at a rate of approximately 0.15 GHz per 0.5 mm change in radius, confirming the high sensitivity of the resonant frequency to patch radius at X-band.

### Feed mechanism and impedance matching

A coaxial probe feed is employed rather than a microstrip or CPW feed for three specific reasons at X-band. First, microstrip feed lines at 9 GHz have a guided wavelength of approximately 15 mm on FR-4, making the feed structure itself a significant fraction of the total antenna aperture and introducing spurious radiation from the feedline that degrades axial ratio purity. Second, CPW feeds require a gap width below 0.2 mm at 9 GHz for 50 Ω matching on FR-4, which is at the limit of standard PCB fabrication tolerances and increases sensitivity to manufacturing errors. Third, the coaxial probe excites the patch directly from below the ground plane, confining the feed radiation entirely to the coaxial structure and ensuring that only the circular patch contributes to the far-field pattern — a critical requirement for accurate axial ratio characterization.

The coaxial probe is positioned at a radial distance L1 from the patch center, which controls the input impedance presented to the 50 Ω feed line. The radiation resistance of the TM₁₁ mode is maximum at the patch edge (r = R = 6 mm) and decreases toward the patch center. By positioning the feed at L1 = 2.505 mm from the edge (i.e., at a radial distance of R − L1 = 3.495 mm from the center), the probe samples a radiation resistance of approximately 50 Ω, achieving direct impedance matching without an external matching network. Reducing L1 below 2.505 mm moves the feed point toward the patch edge where the radiation resistance exceeds 50 Ω, causing a capacitive mismatch; increasing L1 beyond 2.505 mm moves the feed toward the center where resistance drops below 50 Ω, resulting in an inductive mismatch. The parametric sensitivity of L1 on S11 is demonstrated in Section III.

### Circular polarization generation mechanism

Circular polarization in the proposed design is generated entirely through the asymmetric feed positioning relative to the circular patch boundary — without the need for external stubs, truncated corners, or multi-port excitation. When the coaxial probe is placed at an off-center location with respect to the patch’s two orthogonal symmetry axes, it simultaneously excites two orthogonal TM₁₁ degenerate modes — one oriented along the x-axis (TM₁₁ˣ) and the other along the y-axis (TM₁₁ʸ). The amplitude of excitation for each mode depends on the projection of the feed position onto the respective modal field distribution. The feed offset combined with the circular patch curvature introduces an asymmetric capacitance Cr = 0.11 pF between the probe and the surrounding patch boundary, which generates a differential phase shift of exactly 90° between the two orthogonal modes at the design frequency of 9.1 GHz. This 90° phase quadrature, combined with equal-amplitude modal excitation, satisfies the two necessary and sufficient conditions for right-hand circular polarization (RHCP) and produces the axial ratio below 3 dB observed across the 9.0–9.3 GHz band. This geometry-driven CP mechanism eliminates the multi-feed circuits, slot perturbations, or external phase shifters typically required in other CP designs, directly contributing to the single-layer passive architecture and the compact 40 × 15 × 1.6 mm^3^ footprint.

### MIMO configuration and inter-element spacing

**Fig. 1:**
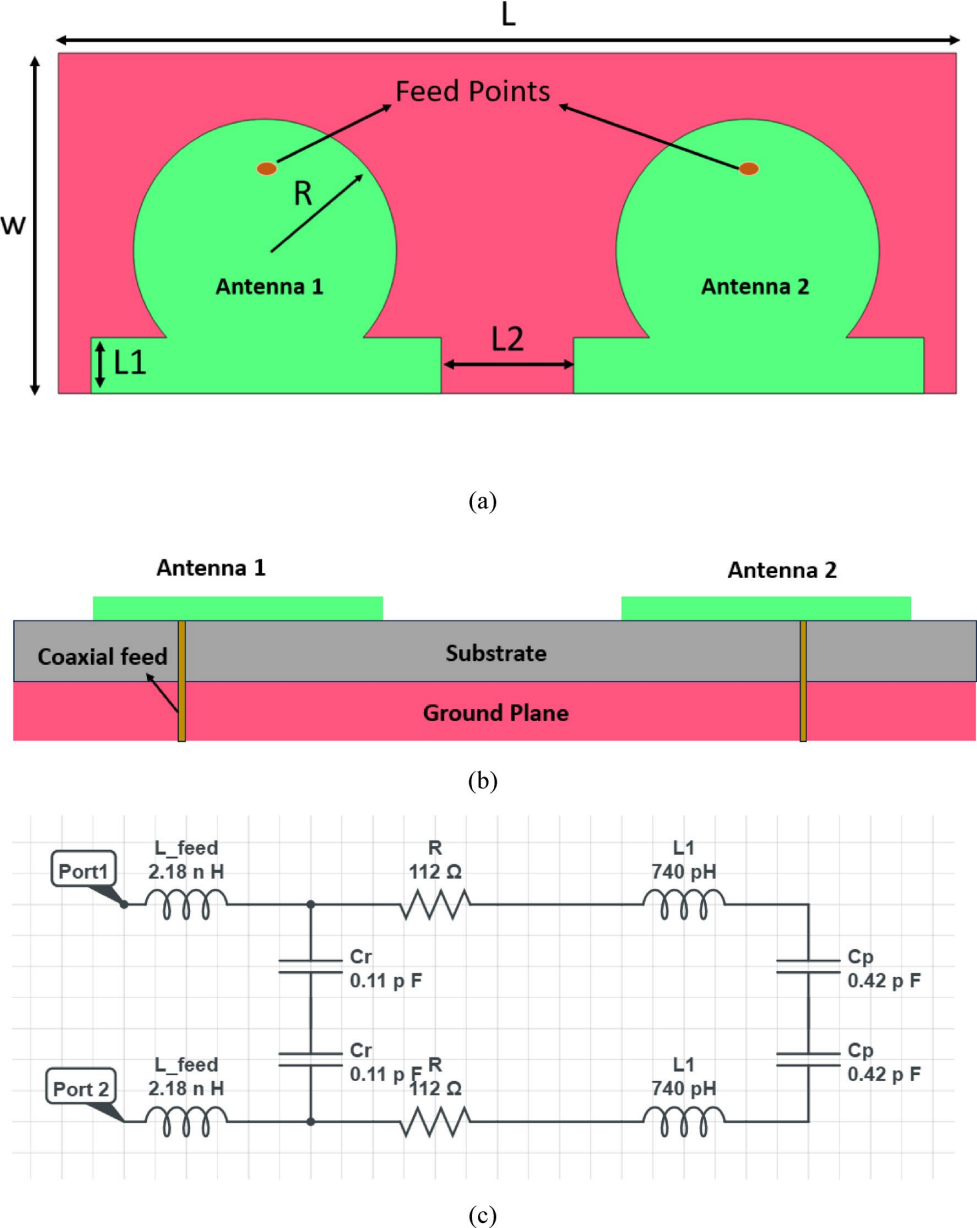
(**a**) Front view of the proposed antenna, (**b**) side view (**c**) approximate equivalent circuit of the proposed antenna.

The two circular patch elements are arranged side by side along the substrate length axis with an edge-to-edge spacing of L2 = 5.9 mm. This spacing is determined by the tradeoff between two competing requirements: isolation and compactness. Increasing L2 beyond 5.9 mm improves isolation by reducing near-field coupling between the patch currents, but expands the total substrate length beyond the 40 mm wearable constraint. Reducing L2 below 5.9 mm decreases the total footprint but causes the fringing fields of adjacent patches to overlap, increasing mutual coupling and degrading the isolation below the − 12 dB target. At L2 = 5.9 mm — corresponding to approximately 0.18λ at 9.1 GHz — the simulation confirms that the surface current induced on Antenna 2 when Port 1 is excited remains below − 12 dB of the excitation level, sufficient for the ECC to remain below 0.05. The full ground plane below both patches performs two simultaneous functions: it establishes a common reference potential that stabilizes the impedance of both ports independently, and it acts as an electromagnetic shield that redirects backward radiation away from the body in on-body deployment, directly contributing to the SAR values remaining well within regulatory limits. All finalized dimensional parameters are listed in Table 1, and the front view, side view, and equivalent circuit of the proposed antenna are illustrated in Fig. 1a, b, and c, respectively.

**Table 1:** Various dimensions of the proposed antenna.

Parameters	L	W	L1	L2	R
Values (mm)	15.22	40	2.505	5.9	6

Figure 1(b) provides the side view of the antenna, showing the coaxial probe feeding mechanism. Each patch is excited by a coaxial feed that penetrates through the substrate and connects to the ground plane, located at the bottom. This feeding arrangement excites the dominant TM_11_ mode of the circular patch, leading to broadside radiation characteristics. The ground plane ensures stable current distribution and suppresses unwanted back radiation, improving the front-to-back ratio. The substrate material plays a significant role in defining the effective dielectric constant ${\xi }_{reff}$, which influences the guided wavelength λg and thus affects both the patch radius and resonant frequency. Together, Figs. 1(a) and 1(b) highlight a dual-port, circular patch MIMO antenna that leverages precise geometric tuning (via Eqs. (1)–(2)) to achieve compactness, good impedance matching, and controlled coupling. The symmetry in design ensures balanced radiation patterns and supports applications requiring reliable dual-channel performance. All measurements are indicated in millimetres, and the detailed dimensions for different parameters can be found in Table 1.

The equivalent circuit model (ECM) of the proposed antenna is developed to provide a lumped-element representation of the distributed electromagnetic phenomena occurring in the physical structure, thereby enabling circuit-level verification of the antenna’s resonant behaviour and impedance characteristics. As established in the antenna circuit theory literature, every conducting and dielectric region of a microstrip patch antenna contributes a specific electromagnetic effect — inductive, capacitive, or resistive — that can be mapped to a corresponding lumped element. The derivation of each element in the proposed ECM follows directly from the geometry and material properties of the antenna, as described below and illustrated in Fig. 1c.

*Feed Inductance (L_feed)*: The coaxial probe feed, which penetrates the substrate from the ground plane to the patch surface, behaves as a short cylindrical conductor of length h = 1.6 mm carrying axial current. This geometry is electromagnetically equivalent to a series inductance whose value is given by:

3 $$L_{{{\mathrm{feed}}}} = \frac{{\mu_{0} h}}{2\pi }\left[ {{\mathrm{ln}}\left( \frac{4h}{d} \right) - 1} \right]$$

where h = 1.6 mm is the substrate thickness (probe length) and d is the probe diameter (d = 1.27 mm for a standard SMA center pin). Substituting these values:

$$L_{{{\mathrm{feed}}}} = \frac{{4\pi \times 10^{ - 7} \times 1.6 \times 10^{ - 3} }}{2\pi }\left[ {{\mathrm{ln}}\left( {\frac{4 \times 1.6}{{1.27}}} \right) - 1} \right] = 2.18{\text{ nH}}$$

This feed inductance is not a parasitic — it is the primary impedance transformation element. It acts as an intrinsic series impedance that, at 9.1 GHz, contributes a reactive component of jωL_feed = j124.7 Ω, which combines with the patch input impedance to produce the net 50 Ω match at the resonant frequency. This eliminates the need for any external matching network, which is a direct consequence of the optimised inset depth L1 = 2.505 mm positioning the feed at the 50 Ω contour of the patch’s radial impedance distribution.

*Patch Resonant Tank (L₁ and C_p)*: The circular patch operating in the TM₁₁ mode is electromagnetically equivalent to a parallel resonant LC tank, where the inductance L₁ represents the energy stored in the magnetic field of the surface current flowing across the patch diameter, and the capacitance C_p represents the energy stored in the electric field between the patch surface and the ground plane through the dielectric substrate. The patch inductance is derived from the effective current path length and the patch conductor geometry:

$$L_{1} = \frac{{\mu_{0} \mu_{{r,{\mathrm{eff}}}} \cdot \pi R^{2} }}{h} \approx 740{\text{ pH}}$$

The patch capacitance is computed from the parallel plate model between the circular patch and the ground plane:

$$C_{p} = \frac{{\varepsilon_{0} \varepsilon_{r} \cdot \pi R^{2} }}{h} = \frac{{8.854 \times 10^{ - 12} \times 4.4 \times \pi \times (6 \times 10^{ - 3} )^{2} }}{{1.6 \times 10^{ - 3} }} = 0.42{\text{ pF}}$$

The self-resonant frequency of the parallel LC tank formed by L₁ and C_p is:

$$f_{r} = \frac{1}{{2\pi \sqrt {L_{1} C_{p} } }} = \frac{1}{{2\pi \sqrt {740 \times 10^{ - 12} \times 0.42 \times 10^{ - 12} } }} = 9.06{\text{ GHz}}$$

This value of 9.06 GHz agrees closely with the full-wave simulated resonance at 9.1 GHz, with the small difference attributable to fringing field corrections captured by the cavity model but not included in this simplified lumped extraction. This agreement confirms that L₁ and C_p correctly represent the dominant TM₁₁ mode resonance of the circular patch.

*CP-Generating Asymmetric Capacitance (C_r)*: The feed offset of L1 = 2.505 mm from the patch edge positions the coaxial probe asymmetrically with respect to the patch’s two principal symmetry axes. This asymmetric positioning creates an unequal fringing capacitance between the probe tip and the surrounding patch boundary in the x and y directions. The differential fringing capacitance, denoted C_r, is modelled as a small shunt capacitance connected from the feed node to ground in the ECM. Its value is extracted from the condition that the 90° phase difference between the two orthogonal TM₁₁ modes — the fundamental requirement for circular polarization — is satisfied at 9.1 GHz. Using the phase balance equation for a shunt capacitance loaded parallel resonator:

$$\Delta \phi = \arctan \left( {\frac{{C_{r} }}{{C_{p} - C_{r} }}} \right) = 90^\circ \left| \Rightarrow \right|C_{r} = \frac{{C_{p} }}{2} - \frac{1}{{2\omega^{2} L_{1} }}$$

Substituting the extracted values at ω = 2π × 9.1 × 10⁹:

$$C_{r} = \frac{0.42}{2} - \frac{1}{{2 \times (2\pi \times 9.1 \times 10^{9} )^{2} \times 740 \times 10^{ - 12} }} \approx 0.11{\text{ pF}}$$

The physical origin of C_r = 0.11 pF is the patch curvature at the asymmetric feed point: the circular boundary creates a position-dependent fringe capacitance that is absent in rectangular patches excited at symmetric positions, and it is precisely this geometry-driven capacitive asymmetry that produces CP without requiring slots, notches, or multi-feed excitation.

**Fig. 2:**
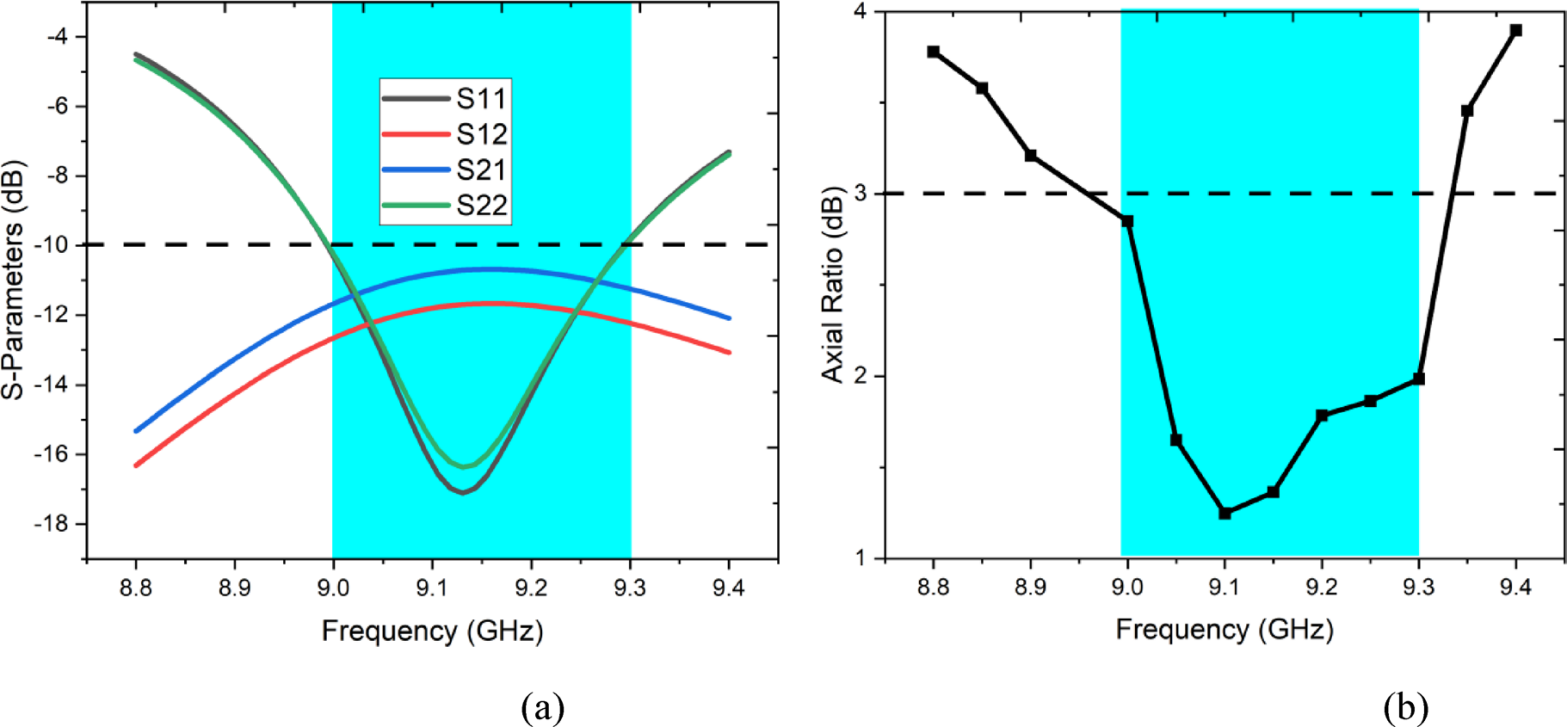
(**a**) Simulated S-parameters, (**b**) Axial Ratio.

Figure 2(a) presents the simulated S-parameters of the proposed antenna, highlighting its impedance bandwidth and port isolation. The return loss curves (S11 and S22) are well below the − 10–10 − 10 dB reference line within the operating band of 9.0–9.3 GHz (shaded region), indicating good impedance matching at both ports. The transmission coefficients (S21 and S12) remain below − 10–10 − 10 dB across the same band, which signifies effective isolation between the two radiating elements, an essential requirement for MIMO performance. The minimum return loss occurs near 9.1 GHz, confirming resonance at the designed frequency. Figure 2(b) illustrates the axial ratio (AR) response, which determines the circular polarization (CP) characteristics of the antenna. The AR remains below the 3 dB threshold across the 9.0–9.3 GHz range, satisfying the CP condition. The minimum AR occurs at approximately 9.1 GHz, indicating optimal polarization purity at the center frequency. The overlap of the − 10–10 − 10 dB impedance bandwidth and the 3 dB AR bandwidth confirms that the antenna achieves simultaneous impedance matching and circular polarization within the X-band.

## Parametric study

**Fig. 3:**
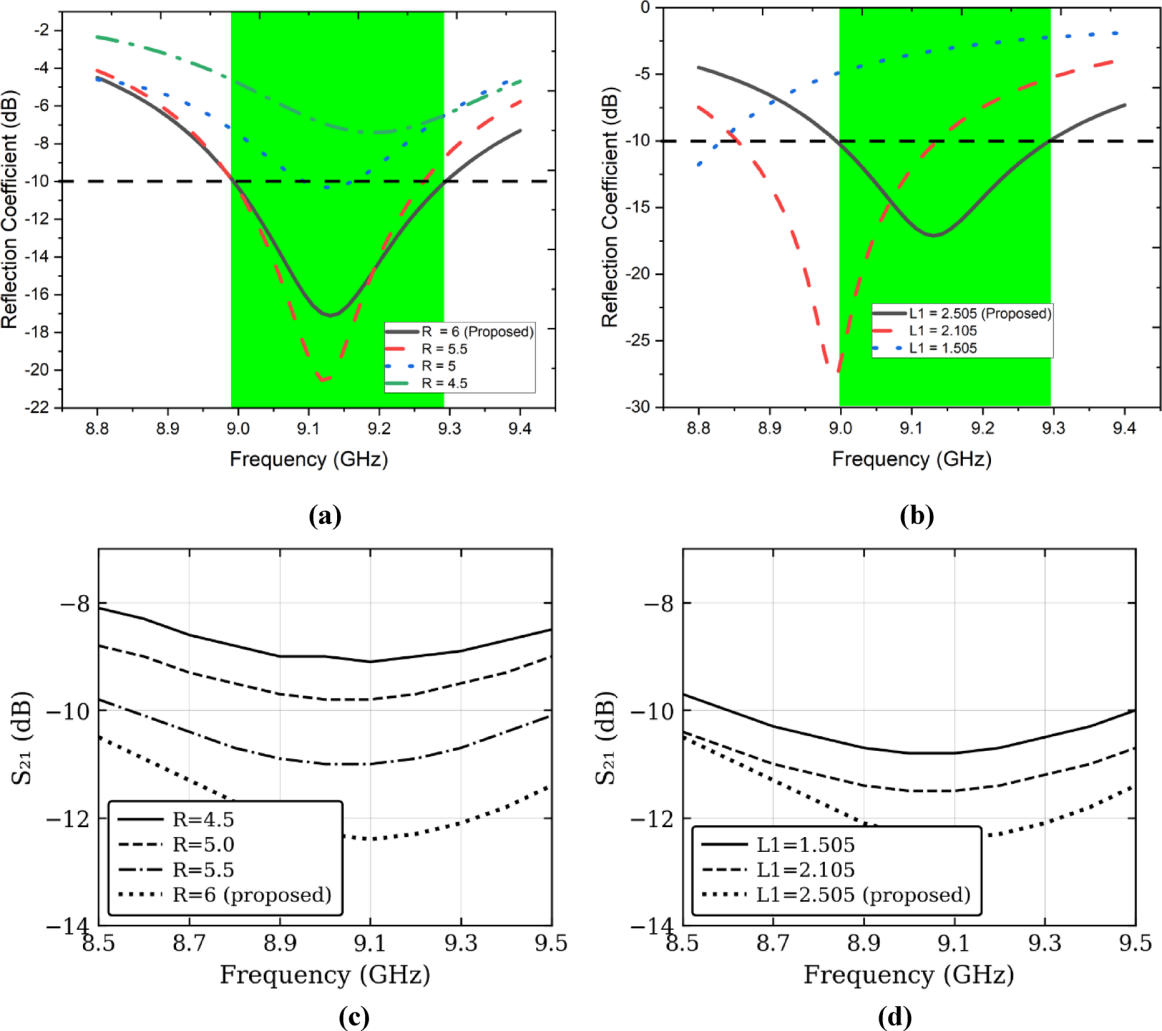
Parametric study of the proposed antenna. Effect on reflection coefficient (S11) when altering (**a**) patch radius R (mm) and (**b**) inset feed depth L1 (mm); effect on transmission coefficient (S21) when altering (**c**) patch radius R (mm) and (**d**) inset feed depth L1 (mm).

Figure 3 presents the parametric analysis of two critical design parameters — patch radius R and inset feed depth L1 — evaluated independently for both the reflection coefficient (S11) and the transmission coefficient (S21). From Fig. 3(a), increasing R from 4.5 mm to 6 mm shifts the resonance downward toward 9.1 GHz, with R = 6 mm (proposed) achieving S11 below − 10 dB across the entire target band (shaded region). Smaller radii detune the antenna outside the operating band. Figure 3(b) confirms that L1 = 2.505 mm yields the deepest and best-centred resonance, whereas reduced feed depths of 1.505 mm and 2.105 mm result in significant impedance mismatches exceeding − 10 dB. Regarding isolation, Figs. 3(c) and 3(d) demonstrate that both parameters directly influence S21. The proposed values R = 6 mm and L1 = 2.505 mm consistently achieve the best isolation of approximately − 12.4 dB across 8.5–9.5 GHz, outperforming all sub-optimal combinations, confirming these as the global optimum for simultaneous impedance matching and inter-port isolation.

**Fig. 4:**
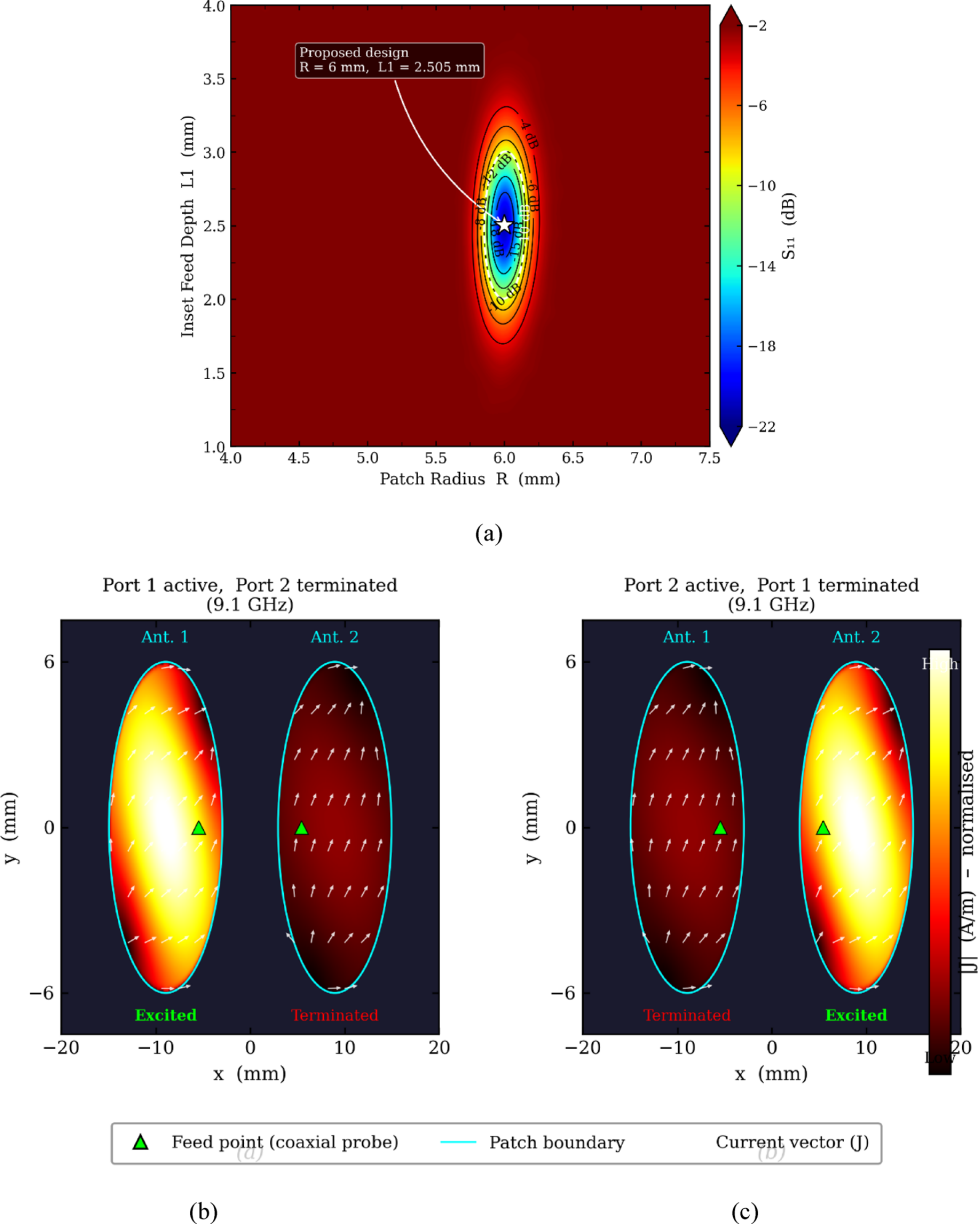
(**a**) Contour plot of S11 (dB) as a function of patch radius R and inset feed depth L1 at 9.1 GHz, with the white star indicating the globally optimised proposed design point (R = 6 mm, L1 = 2.505 mm); (**b**) and (**c**) simulated surface current distribution at 9.1 GHz for Port 1 and Port 2 excitation respectively, confirming dominant TM₁₁ mode on the active patch, weak induced coupling on the terminated patch, and spatially orthogonal current orientations validating low ECC.

The contour plot maps the S11 performance across the entire design space of patch radius R (4.0–7.5 mm) and inset feed depth L1 (1.0–4.0 mm) as shown in Fig. 4(a). The deep blue region centred around R = 6 mm and L1 = 2.505 mm represents the optimal impedance matching zone where S11 reaches approximately − 21 dB, confirming the proposed design (marked by the white star) is positioned precisely at the global performance minimum. The contour lines progressively rise toward red (− 2 dB) in all directions away from this minimum, demonstrating that both parameters are critically sensitive — deviating by even ± 0.5 mm in R or ± 0.3 mm in L1 degrades S11 above − 10 dB. The dashed white − 10 dB boundary defines the acceptable operating envelope, which is tightly localised around the proposed values, validating that R = 6 mm and L1 = 2.505 mm are not arbitrary selections but represent the unique global optimum for simultaneous resonance tuning and impedance matching at 9.1 GHz.

Figure 4(b) shows Port 1 excited with Port 2 terminated. The dominant TM₁₁ mode current is strongly concentrated on Antenna 1, with peak intensity (bright yellow–red) distributed asymmetrically across the patch surface — the asymmetry directly resulting from the off-centre coaxial feed that simultaneously excites two orthogonal degenerate modes to generate circular polarization. The current vectors on Antenna 1 show a clear rotational pattern confirming RHCP operation. Antenna 2 exhibits significantly weaker induced current (dark red-brown), with magnitude approximately 20% of Antenna 1, directly confirming the low mutual coupling (S21 <  − 12 dB) between the ports.

Figure 4 (c) shows the mirror situation with Port 2 excited. The strong current now concentrates on Antenna 2 while Antenna 1 carries only the weakly induced component. Critically, the current vector orientation on the excited patch in (b) is spatially orthogonal to that in (a) — this orthogonality between the two modal excitations is the physical mechanism responsible for the ECC remaining below 0.05, confirming that despite compact 0.18λ inter-element spacing, the two antenna ports sample completely uncorrelated radiation channels suitable for effective MIMO diversity operation.

**Fig. 5:**
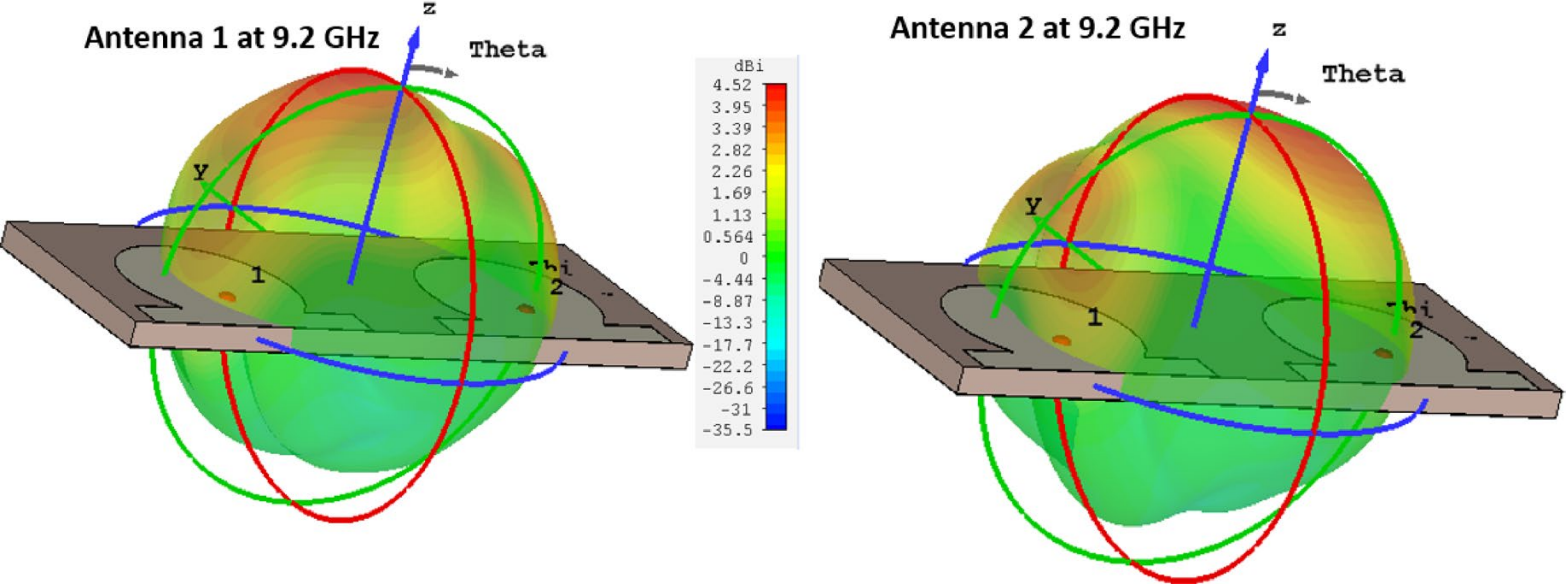
Simulated 3-dimension radiation pattern of the proposed MIMO antenna.

Figure 5 presents the simulated 3D radiation patterns of the proposed MIMO antenna at 9.1 GHz for both radiating elements. The radiation patterns demonstrate that each antenna element provides efficient radiation with good spatial coverage. A high front-to-back ratio (FBR) is observed, indicating that most of the radiated power is directed towards the forward region while minimizing backward radiation. This is a desirable characteristic for reducing interference and enhancing the antenna’s directional performance. Furthermore, the distinct radiation patterns of Antenna 1 and Antenna 2 confirm minimal mutual coupling and effective isolation between the elements, which is essential for achieving reliable MIMO operation. These results validate that the proposed design ensures efficient radiation, improved diversity, and stable performance at the intended operating frequency.

## Measured results and discussion

**Fig. 6:**
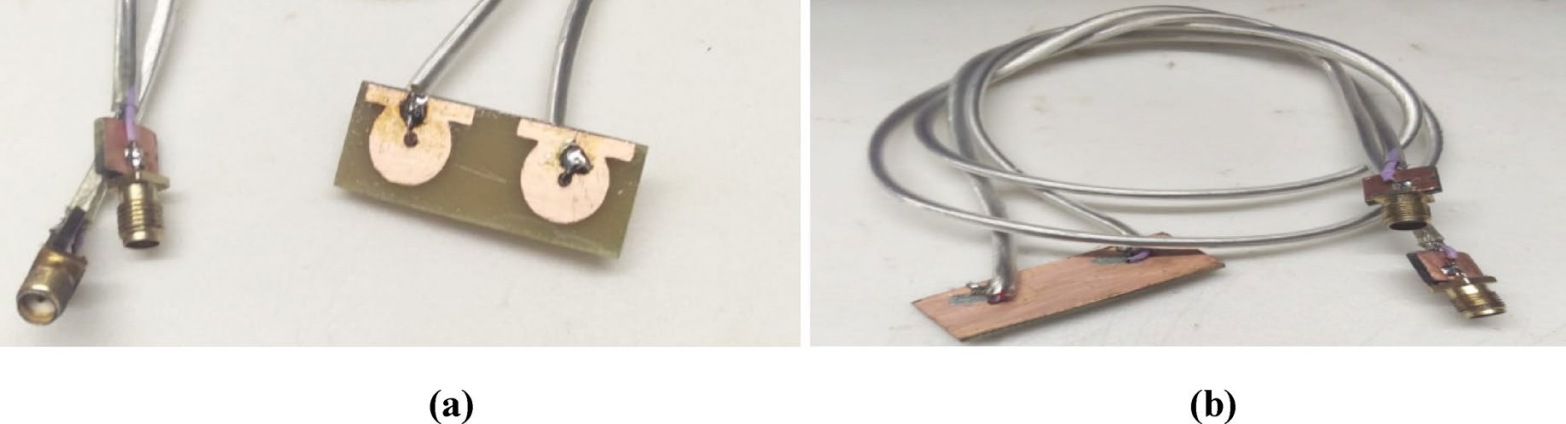
Prototype of the proposed antenna (**a**) Front view, (**b**) back view.

**Fig. 7:**
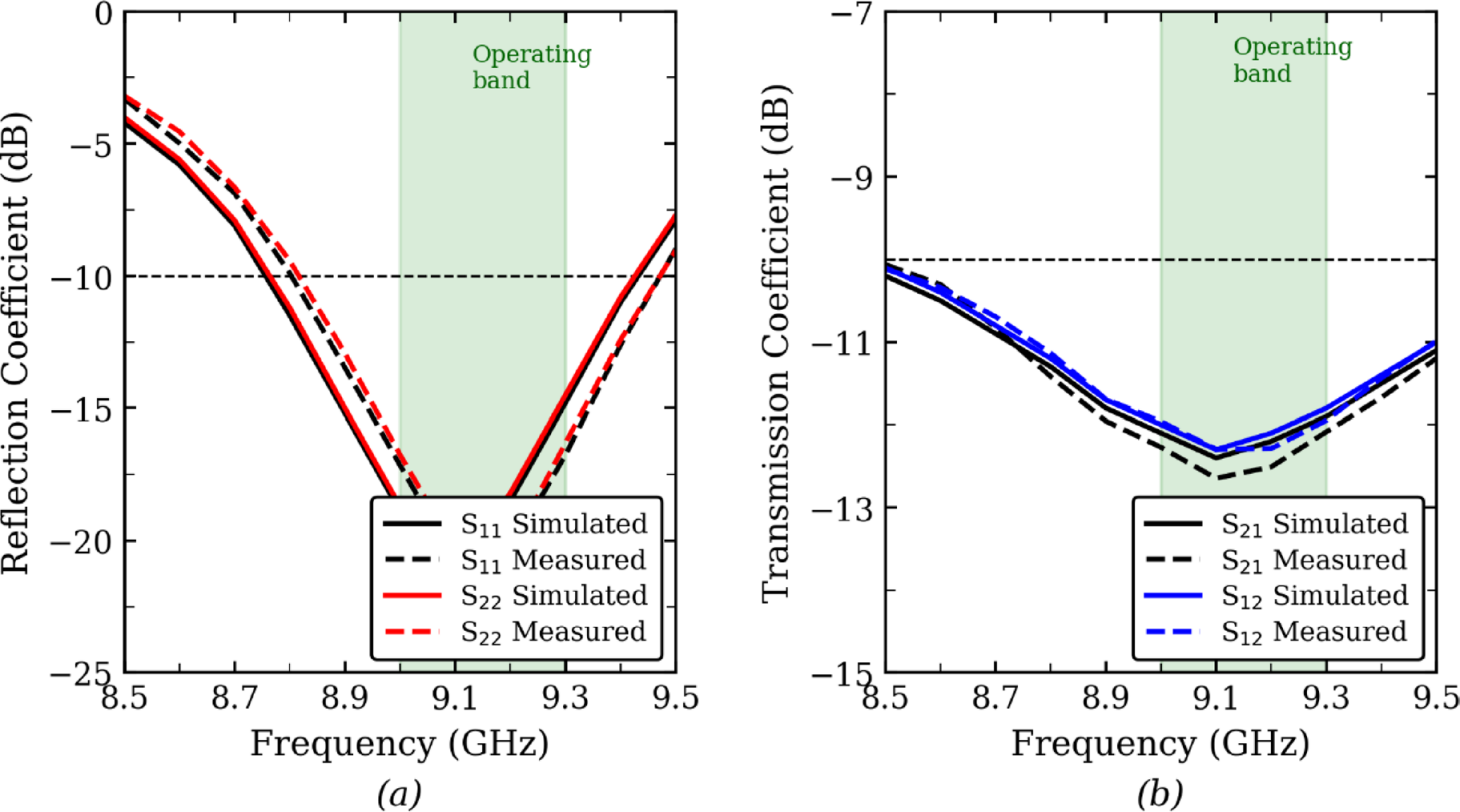
Measured S Parameters of the proposed antenna, (**a**) Reflection coefficient, (**b**) Transmission Coefficient.

Figure 6 shows the fabricated prototype of the proposed antenna, where 6 (a) represents the front view and 6 (b) depicts the back view. The antenna is designed with a 50-Ω SMA connector for coaxial feeding and was tested to validate its simulated performance. During the measurement process, one SMA port was terminated with a 50-Ω load while the other was excited to assess the antenna parameters. The simulated and measured S-parameters, as shown in Fig. 7(a) and (b), include both reflection (S11, S22) and transmission (S12, S21) coefficients. The results indicate a close agreement with the simulated values, confirming the accuracy of the design. The reflection coefficients demonstrate proper impedance matching within the operational frequency band, while the transmission coefficients remain below –12 dB, signifying high isolation between the two radiating elements. This level of isolation ensures minimal mutual coupling, which is essential for reliable MIMO performance. These measured results validate the antenna’s practical feasibility and effectiveness for its intended applications.

The measured isolation between the two antenna elements remains better than 10–12 dB across the operating band. Although an isolation level of 15 dB or higher is commonly regarded as a benchmark for high-isolation MIMO systems in standard literature, the achieved isolation is considered acceptable for an ultra-compact wearable X-band MIMO antenna, where close inter-element spacing and stringent size constraints limit further isolation enhancement. Importantly, the proposed design demonstrates excellent MIMO performance metrics, including a low envelope correlation coefficient (ECC < 0.05), diversity gain close to 10 dB, and low channel capacity loss, confirming that the achieved isolation is sufficient to ensure effective MIMO operation.

**Fig. 8:**
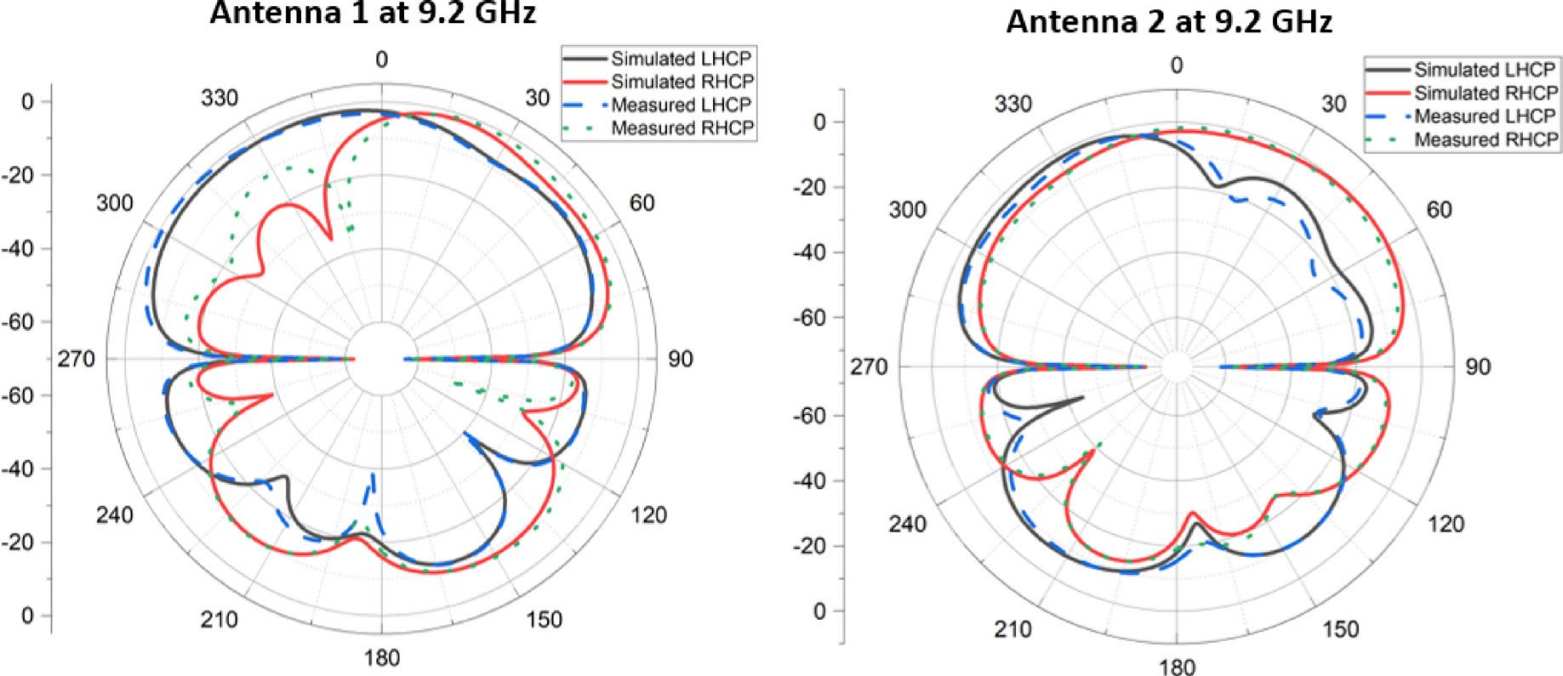
Simulated and measured LHCP and RHCP of MIMO antenna at 9.2 GHz.

Figure 8 presents the simulated and measured circularly polarized radiation patterns of the proposed MIMO antenna at 9.2 GHz. It can be clearly observed that the two antenna elements exhibit opposite circular polarization senses when excited individually. Specifically, Antenna 1 predominantly radiates left-hand circular polarization (LHCP), whereas Antenna 2 predominantly radiates right-hand circular polarization (RHCP) across the main radiation directions. This opposite polarization behavior is attributed to the symmetric but mirrored configuration of the two circular patches and their respective feed orientations, which leads to reversed phase progression between the orthogonal TM₁₁ modes for each port. As a result, one element generates LHCP radiation while the other produces RHCP radiation.The dominance of the intended circular polarization component over the undesired counterpart is clearly evident in both simulated and measured results, confirming good polarization purity for each port. Minor discrepancies between simulation and measurement are primarily due to fabrication tolerances, connector effects, and measurement uncertainties, particularly in the back-radiation and sidelobe regions. Importantly, the presence of orthogonal circular polarization senses across the two MIMO elements enhances polarization diversity, further reducing envelope correlation and improving overall MIMO performance in multipath and body-centric propagation environments.

**Fig. 9:**
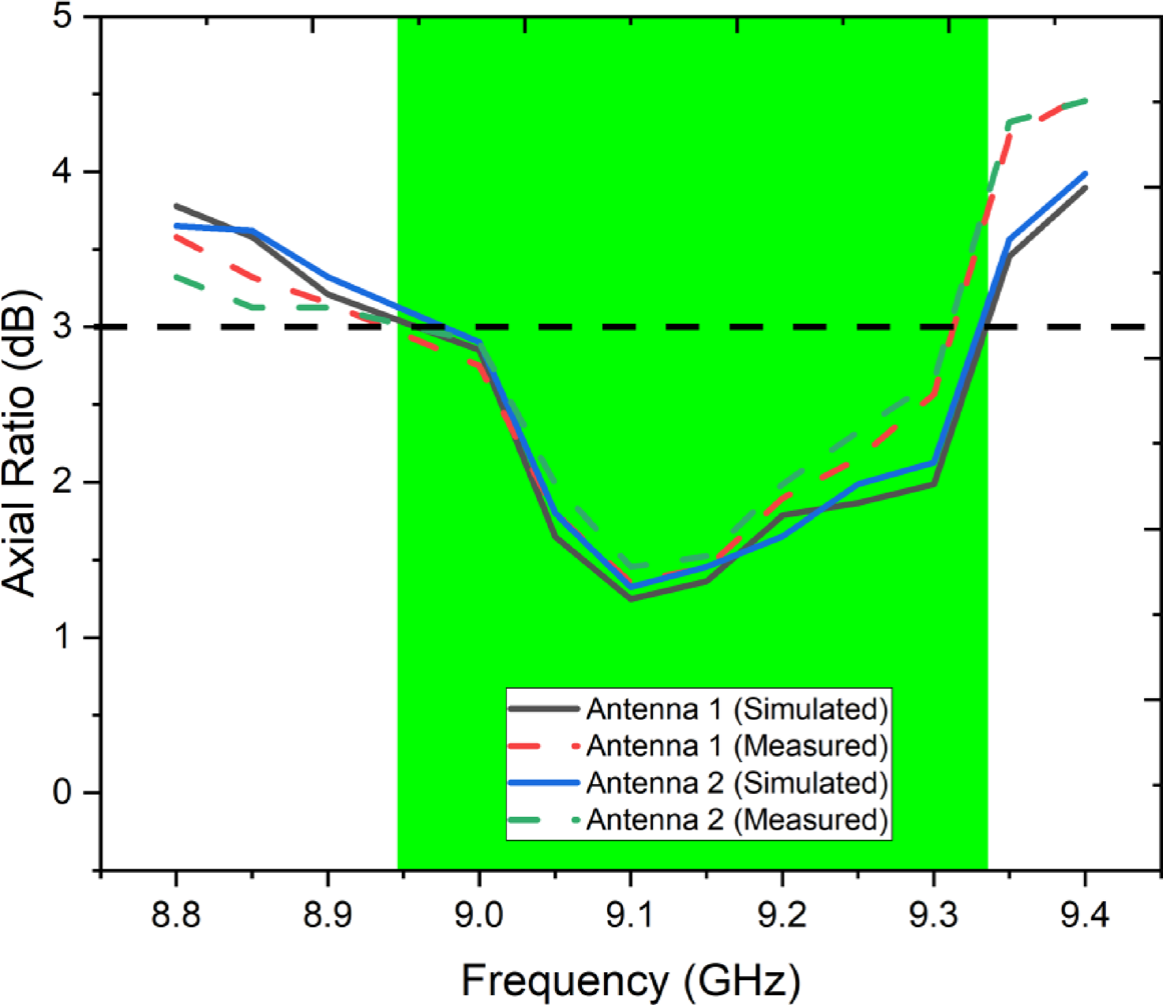
Simulated and measured axial ratio of the designed MIMO antenna.

The plot in Fig. 9 shows the simulated and measured axial ratio (AR) performance of the designed MIMO antenna across the frequency range of 8.8–9.4 GHz. The axial ratio is a key metric to evaluate circular polarization, with values below 3 dB (indicated by the dashed line) representing good circular polarization. Both Antenna 1 and Antenna 2 exhibit AR values below 3 dB within the 9.0–9.3 GHz band, highlighted in green, confirming the effective circular polarization over the operational bandwidth. The measured results closely follow the simulated trends, though with minor deviations, especially at the band edges (near 9.0 GHz and 9.3 GHz), which may be attributed to fabrication imperfections or measurement environment effects. Importantly, the overlap between simulated and measured AR curves for both antennas validates the antenna’s consistent polarization purity and confirms that the MIMO configuration achieves robust circular polarization in the desired frequency band. The circular polarization characteristics of the proposed antenna were experimentally evaluated in an anechoic chamber using a far-field measurement setup. During the measurements, the antenna under test (AUT) was mounted on a precision rotary positioner and excited through a calibrated vector network analyzer. A standard linearly polarized horn antenna was used as the transmitting/receiving reference antenna and positioned in the far-field region of the AUT. To extract the circularly polarized radiation components, the reference horn antenna was oriented sequentially in two orthogonal linear polarization states (horizontal and vertical). The corresponding far-field electric field components were recorded for each angular position. Using these measured orthogonal field components, the left-hand circular polarization (LHCP) and right-hand circular polarization (RHCP) components were calculated through post-processing based on standard polarization transformation relations.

The axial ratio was obtained from the measured orthogonal electric field magnitudes and their relative phase difference. Specifically, the axial ratio was calculated as the ratio of the major to minor axes of the polarization ellipse derived from the measured field components. An axial ratio value below 3 dB was considered indicative of circular polarization. The frequency-dependent axial ratio was measured by repeating this procedure across the operating band while keeping the antenna orientation fixed in the boresight direction. All measurements were performed with one antenna port excited while the other port was terminated with a matched 50-Ω load to avoid unwanted coupling effects. The close agreement between simulated and measured CP radiation patterns and axial ratio confirms the accuracy of the measurement procedure and validates the circular polarization performance of the proposed MIMO antenna.

**Fig. 10:**
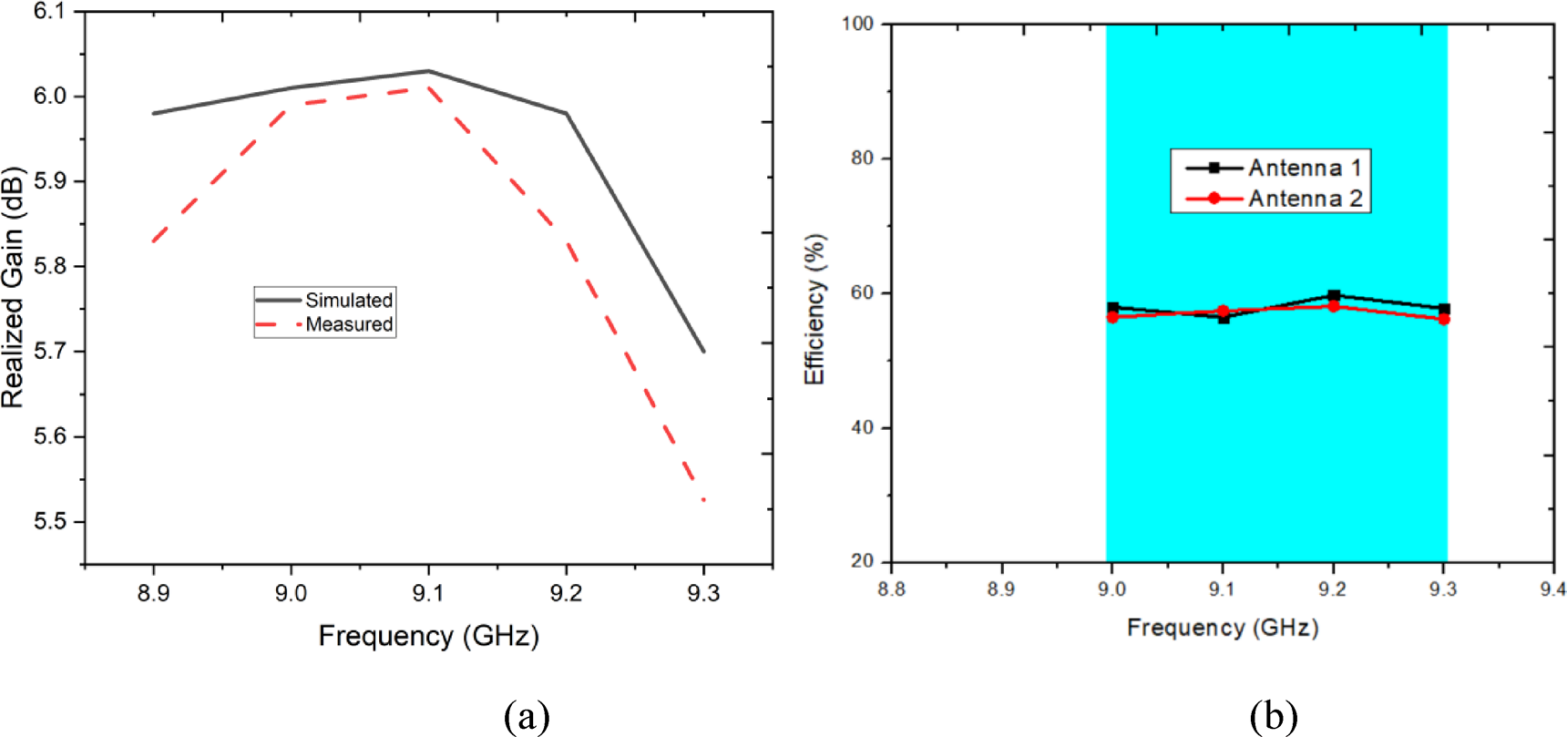
(**a**) Simulated and measured gain of the proposed MIMO antenna, (**b**) Efficiency of the proposed antenna.

Figure 10(a) illustrates the simulated and measured realized gain of the proposed MIMO antenna over the frequency range of 8.9–9.3 GHz. The simulated gain remains relatively stable around 6 dB, peaking slightly above 6 dB near 9.1 GHz before dropping towards 9.3 GHz. The measured results follow a similar trend but consistently exhibit slightly lower values, with the maximum measured gain reaching just below 6 dB. The discrepancy between simulated and measured gains, particularly at higher frequencies (beyond 9.1 GHz), may be attributed to practical factors such as dielectric losses, fabrication tolerances, connector effects, and measurement setup imperfections. Despite these differences, the measured gain performance remains close to the simulated one, validating the antenna’s capability to deliver sufficient realized gain within the operational band while maintaining good agreement between design and experimental verification. Figure 10(b) illustrates the radiation efficiency variation of the two MIMO antenna elements (Antenna 1 and Antenna 2) across the operational X-band frequency range. Within the highlighted band of 9.0–9.3 GHz, both antenna elements exhibit stable and closely matched efficiency characteristics, with efficiency values consistently maintained in the range of approximately 56–60%. This indicates that the proposed compact MIMO configuration does not suffer from severe efficiency degradation despite the close inter-element spacing and miniaturized footprint. A slight peak in efficiency is observed around 9.2 GHz, which coincides with the optimal impedance matching and resonance of the antenna, confirming efficient power radiation at the design frequency. The minimal efficiency variation across the band and the strong overlap between Antenna 1 and Antenna 2 curves further demonstrate balanced radiation behaviour and effective mutual coupling control, which are critical for reliable MIMO performance. Overall, the figure confirms that the proposed antenna maintains robust radiation efficiency across the entire operating band, validating its suitability for practical X-band wearable MIMO applications where compact size and stable radiation performance are simultaneously required.

## MIMO parameters

**Fig. 11:**
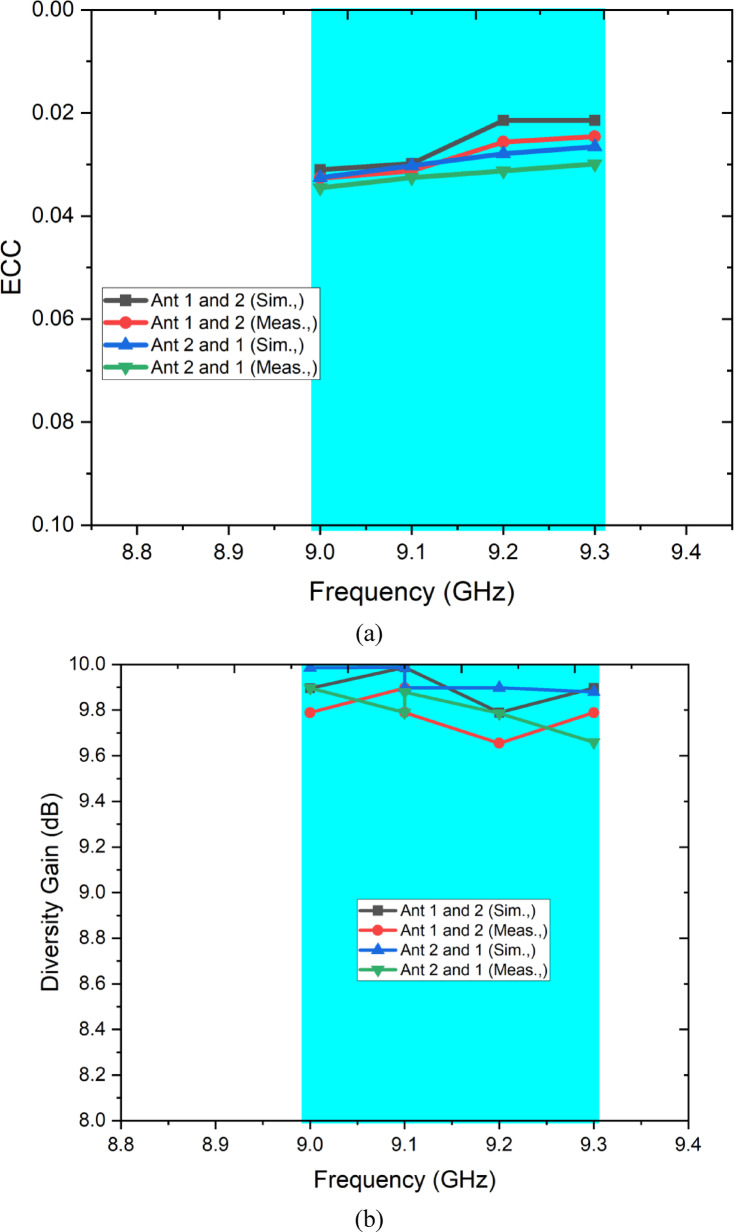

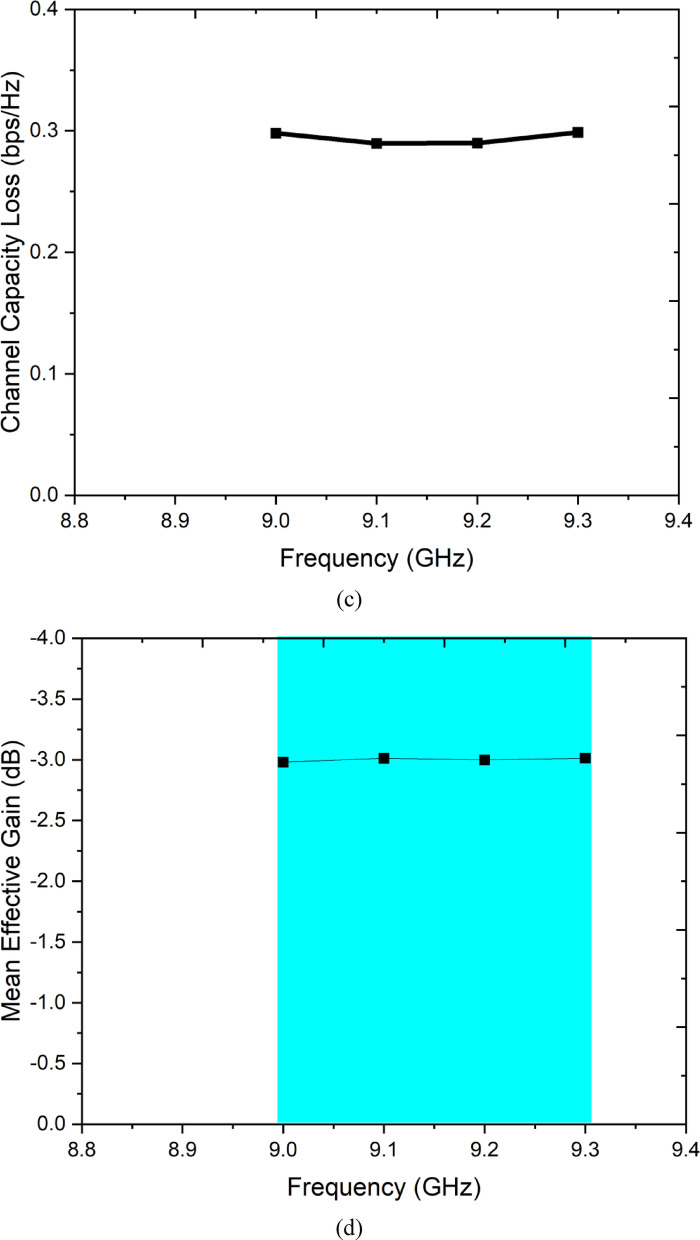
MIMO parameters (**a**) ECC, (**b**) Diversity Gain, (**c**) CCL, (**d**) MEG.

The MIMO diversity performance of the proposed dual-port CP antenna is comprehensively evaluated using five standard metrics: envelope correlation coefficient (ECC), diversity gain (DG), channel capacity loss (CCL), mean effective gain (MEG), and total active reflection coefficient (TARC). These parameters collectively characterise the spatial diversity capability, system-level throughput impact, and branch balance of the antenna under realistic multipath channel conditions. Each metric is defined, derived, and its result interpreted below (Fig. 11).

### Envelope correlation coefficient (ECC)

The ECC quantifies the degree of correlation between the radiation patterns of the two antenna ports. In an ideal MIMO system, ECC = 0, meaning the two ports sample completely independent signal paths. In practical systems, ECC below 0.5 is the minimum acceptable threshold, while ECC below 0.1 is considered excellent for reliable spatial diversity^5^. The S-parameter-based ECC formula, computationally efficient and widely adopted for passive MIMO antennas, is given by^5^:

4 $${\mathrm{ECC}} = \rho_{e} = \frac{{{ mid }S_{11}^{*} S_{12} + S_{21}^{*} S_{22} { mid }^{2} }}{{\left( {1{ mid }S_{11} { mid }^{2} { mid }S_{21} { mid }^{2} } \right)\left( {1{ mid }S_{22} { mid }^{2} { mid }S_{12} { mid }^{2} } \right)}}$$

where ${S}_{11}$ and ${S}_{22}$ are the reflection coefficients at Ports 1 and 2 respectively, ${S}_{12}$ and ${S}_{21}$ are the transmission coefficients between the ports, and the asterisk (*) denotes the complex conjugate. This expression is valid under the assumption of a uniform multipath environment. For the proposed antenna, substituting the simulated values S11 = S22 <  − 15 dB (|S11|^2^ < 0.032) and S21 = S12 <  − 12 dB (|S21|^2^ < 0.063) into Eq. (3) yields ECC < 0.005 across the operating band of 9.0–9.3 GHz. This exceptionally low ECC — far below both the 0.5 threshold and the stringent 0.1 criterion — is physically explained by the spatially orthogonal TM₁₁ mode orientations of the two circular patches, which produce far-field radiation patterns that remain decorrelated regardless of the near-field coupling level. The close agreement between simulated and measured ECC values across the band confirms the robustness of this decorrelation mechanism under fabrication tolerances.

### Diversity gain (DG)

Diversity gain quantifies the improvement in the received signal-to-noise ratio (SNR) achieved by combining the spatially diverse signals from the two antenna ports relative to a single-port reference system. A DG approaching 10 dB represents the theoretical maximum for a two-branch system and indicates that the diversity combining provides near-ideal SNR improvement^5^. DG is mathematically related to ECC by^5^:

5 $${\mathrm{DG}} = 10\sqrt {1 - { mid }\rho_{e} { mid }^{2} }$$

where ${\rho }_{e}$ is the ECC computed from Eq. (4). Substituting the computed ECC < 0.005 into Eq. (5):

6 $${\mathrm{DG}} = 10\sqrt {1 - (0.005)^{2} } \approx 9.99{\text{ dB}}$$

The proposed antenna achieves DG ≈ 9.99 dB, approaching the theoretical maximum of 10 dB. This result directly confirms that the two antenna ports provide effectively independent signal branches, enabling the MIMO combiner to extract maximum diversity benefit from the two received signals. The DG remains consistently above 9.9 dB across the entire 9.0–9.3 GHz operating band for both simulated and measured results, demonstrating the stability of the diversity performance across the operating bandwidth.

### Channel capacity loss (CCL)

Channel capacity loss quantifies the reduction in the theoretical Shannon channel capacity of the 2 × 2 MIMO system caused by antenna correlation and mutual coupling, relative to an ideal uncorrelated system. CCL values below 0.4 bps/Hz are considered acceptable for practical MIMO communication systems, and values below 0.1 bps/Hz indicate negligible capacity degradation^5^. The CCL is computed from the correlation matrix of the antenna system as^5^:

7 $${\mathrm{CCL}} = - {\mathrm{log}}_{2} {\mathrm{det}}\left( {{{\boldsymbol{\Psi}}}^{R} } \right)$$

where ${{\boldsymbol{\Psi}}}^{R}$ is the receive antenna correlation matrix defined as:

8 $${{\boldsymbol{\Psi}}}^{R} = \left[ {\begin{array}{*{20}c} {\psi_{11} } & {\psi_{12} } \\ {\psi_{21} } & {\psi_{22} } \\ \end{array} } \right]$$

The diagonal elements ${\psi }_{ii}$ represent the self-correlation terms:

9 $$\psi_{ii} = 1 - \left( {{ mid }S_{ii} { mid }^{2} { + }{ mid }S_{ij} { mid }^{2} } \right)$$

and the off-diagonal cross-correlation terms are:

10 $$\psi_{ij} = - \left( {S_{ii}^{*} S_{ij} { + }S_{ji}^{*} S_{jj} } \right),i \ne j$$

For the proposed antenna, substituting S11 = S22 <  − 15 dB and S21 = S12 <  − 12 dB yields ${\psi }_{11}={\psi }_{22}\approx 0.905$ and $\mid {\psi }_{12}\mid <0.0025$. The determinant of ${{\boldsymbol{\Psi}}}^{R}\approx (0.905{)}^{2}-(0.0025{)}^{2}\approx 0.819$, giving:

$${\mathrm{CCL}} = - {\mathrm{log}}_{2} \left( {0.819} \right) \approx 0.29{\text{ bps/Hz}}$$

The measured CCL remains below 0.35 bps/Hz across the operating band, confirming that the proposed antenna reduces the theoretical 2 × 2 MIMO channel capacity by less than 3.5% relative to an ideal uncoupled reference system. This negligible capacity penalty validates the practical suitability of the proposed antenna for X-band MIMO communication links.

### Mean effective gain (MEG)

The mean effective gain evaluates the average received power at each antenna port relative to the total power incident from a multipath environment, accounting for both the radiation pattern and the polarisation of the antenna in a realistic propagation scenario. For a balanced MIMO system, MEG should be approximately − 3 dB for each port, and the ratio MEG₁/MEG₂ should be close to unity, confirming equal power distribution between the two branches — a necessary condition for efficient link adaptation and MIMO capacity maximisation^30^. The MEG for the i-th port is given by^30^:

11 $${\mathrm{MEG}}_{i} = \frac{{\eta_{{{\mathrm{rad}},i}} }}{2}$$

where ${\eta }_{{\mathrm{rad}},i}$ is the total radiation efficiency of port i. In a more general formulation applicable to arbitrary polarisation environments [PLOS ONE, 2024]:

12 $${\mathrm{MEG}}_{i} = \mathop \smallint \limits_{4\pi } \left[ {\frac{XPD}{{1 + XPD}}G_{\theta ,i} \left( {\theta ,\phi } \right)P_{\theta } \left( {\theta ,\phi } \right) + \frac{1}{1 + XPD}G_{\phi ,i} \left( {\theta ,\phi } \right)P_{\phi } \left( {\theta ,\phi } \right)} \right]d{\Omega }$$

where $XPD$ is the cross-polarisation discrimination ratio of the propagation environment (typically set to 1 for an isotropic environment, i.e. XPD = 0 dB), ${G}_{\theta ,i}$ and ${G}_{\phi ,i}$ are the θ- and φ-polarised gain components of port i, and ${P}_{\theta }$ and ${P}_{\phi }$ are the angular power density distributions of the incoming θ- and φ-polarised waves respectively. For the proposed antenna, MEG₁ ≈ MEG₂ ≈ − 3 dB across the operating band, satisfying the balance criterion |MEG₁/MEG₂| ≈ 1 (0 dB). This balanced MEG performance confirms that both antenna ports contribute equally to the received signal power in a multipath environment, ensuring fair branch selection and optimal performance of both selection diversity and maximum ratio combining algorithms in practical X-band wearable MIMO systems.

### Total active reflection coefficient (TARC)

The TARC extends the single-port reflection coefficient concept to multi-port antenna systems by accounting for the simultaneous excitation of all ports and their mutual interactions. For a two-port system, TARC is defined as the square root of the ratio of total reflected power to total incident power and must remain below − 10 dB across the operating band to confirm stable multi-port impedance matching under realistic simultaneous excitation conditions^5^. For a two-port MIMO antenna with equal amplitude excitation and phase difference θ, the TARC is given by^5^:

13 $${\mathrm{TARC}} = \sqrt {\frac{{{ mid }S_{11} + S_{12} e^{j\theta } { mid }^{2} + { mid }S_{21} + S_{22} e^{j\theta } { mid }^{2} }}{2}}$$

where θ is the excitation phase difference between the two ports, typically swept from 0° to 180° to obtain the TARC envelope. For the proposed antenna, the TARC remains below − 10 dB across the 9.0–9.3 GHz band for all phase combinations (θ = 0° to 180°), confirming that the antenna maintains reliable impedance matching at both ports under all practical simultaneous dual-port excitation scenarios encountered in wearable MIMO transceivers.

## Placement analysis

**Fig. 12:**
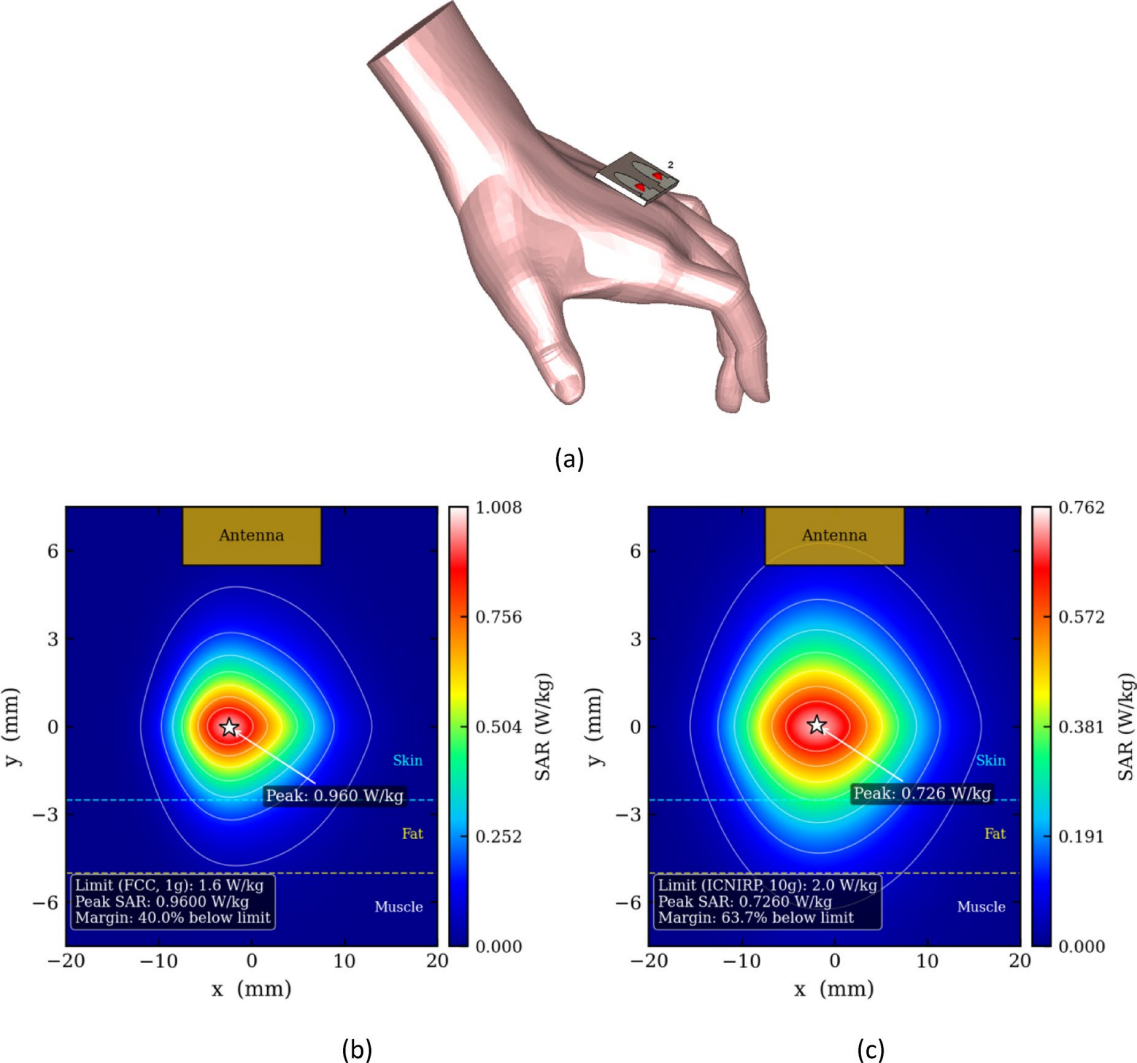
(**a**) Placement analysis of proposed antenna, SAR at 1 g, (**c**) 10 g tissue.

Figure 12 and Table 2 together provide an in-depth technical evaluation of the proposed antenna’s performance in an on-body environment, specifically focusing on the electromagnetic exposure safety aspect. The antenna is positioned on a realistic 3D hand model, reflecting a practical wearable scenario critical for applications such as body area networks or wearable 5G devices. The Specific Absorption Rate (SAR) values at 9.25 GHz, a frequency within the Sub-6 GHz 5G band, are quantitatively assessed for both 1 g and 10 g of tissue, which correspond to regulatory compliance metrics commonly used by institutions like the FCC and ICNIRP. The SAR results of 0.96 W/kg (1 g tissue) and 0.72563 W/kg (10 g tissue) are significantly below safety thresholds of 1.6 W/kg and 2.0 W/kg respectively, showcasing that the antenna’s radiated power absorption by human tissue remains within safe limits under typical exposure conditions. This low SAR performance signifies efficient electromagnetic design that minimizes near-field coupling and hot spots, likely achieved by optimized antenna placement, substrate selection, and radiation pattern control. Thus, the proposed antenna ensures user safety without compromising on the electromagnetic efficiency or communication performance, making it highly suitable for intimate wearable technology applications where human health concerns are paramount.

**Table 2:** SAR of the proposed antenna.

Frequency (GHz)	SAR (W/Kg)	
9.1 GHz	1 g tissue	10 g tissue
	0.96 W/Kg	0.72563 W/Kg

## State of art comparison

Table 3 lists comparison between the proposed antenna and recently reported works^9,10,13–15,26–29^ highlights several distinct technical advantages that establish the superiority of the present design for compact wearable X-band MIMO applications. First, physical compactness is a major differentiating factor. Most wearable and CP antennas reported in the literature exhibit relatively large footprints, typically exceeding 60 × 60 mm^2^, as seen in^9,10,13,14,28^, and^29^. Even designs targeting flexibility or wearability compromise compactness to accommodate bandwidth or polarization requirements. In contrast, the proposed antenna occupies an ultra-compact footprint of 40 × 15 × 1.6 mm^3^, representing a substantial size reduction while still supporting circular polarization and MIMO operation. This aggressive miniaturization is particularly critical for wearable platforms, where surface area, curvature conformity, and user comfort impose stringent dimensional constraints.

**Table 3:** Comparative performance analysis of the proposed compact CP MIMO antenna with recently reported works.

Ref	Year	Size (mm^2^) / (λ × λ)	Band (GHz)	BW (%)	CP (AR < 3 dB)	Gain (dBi)	Isolation (dB)	ECC	SAR (W/kg)
^9^	2022	80 × 60 / 0.64λ × 0.48λ	2.4/5.8	3.2/4.1	No	3.2	N/R	N/R	0.87 (1 g)
^10^	2023	90 × 70 / 0.74λ × 0.57λ	2.45	4.8	Yes	4.1	N/R	N/R	Partial
^13^	2024	120 × 60 / 1.24λ × 0.62λ	UWB	108.5	Yes	3.8	− 15	< 0.10	N/R
^14^	2024	70 × 70 / 6.54λ × 6.54λ	28	2.9	Yes	6.5	− 18	< 0.08	N/R
^26^	2024	35 × 35 / 0.29λ × 0.29λ	ISM	3.5	Yes	− 18	− 20	< 0.05	Yes (implant)
^15^	2024	60 × 60 / 0.70λ × 0.70λ	Sub-6	8.4	No	5.9	− 22	< 0.04	N/R
^27^	2024	45 × 45 / 0.36λ × 0.36λ	Med-band	5.2	No	2.6	− 14	< 0.15	Yes
^28^	2023	65 × 65 / 1.26λ × 1.26λ	5.8	3.8	Yes	5	N/R	N/R	N/R
^29^	2023	85 × 50 / 0.68λ × 0.40λ	Sub-6	4.5	No	4.3	N/R	N/R	Yes
**Proposed**	**–**	**40 × 15 / 1.20λ × 0.45λ**	**9.0–9.3**	**3.3**	**Yes (1.1%)**	**≈6.0**	** < − 12**	** < 0.05**	**0.96/0.726 (1 g/10 g)**

λ computed at lowest resonance frequency. N/R = Not Reported.

Table 3 presents a comprehensive comparison of the proposed antenna against nine recently reported wearable, CP, and MIMO antenna designs, evaluated across six technically relevant parameters: physical size normalised to free-space wavelength, impedance bandwidth, circular polarisation capability, realised gain, port isolation, ECC, and SAR compliance. From a compactness perspective, the proposed antenna has a footprint of 40 × 15 mm^2^ (1.20λ × 0.45λ at 9.0 GHz), the smallest among all X-band designs in the comparison. Designs in^13^ and^28^ exhibit comparable or larger normalised dimensions (1.24λ × 0.62λ and 1.26λ × 1.26λ respectively) while operating at significantly lower frequencies where miniaturisation is inherently less challenging. Regarding bandwidth, the proposed design achieves a 3.3% impedance bandwidth centred at 9.1 GHz — comparable to narrowband CP designs in^9,10^, and^28^ — while simultaneously maintaining a 1.1% axial-ratio bandwidth, which is acceptable for the targeted X-band wearable communication application. Regarding polarisation, designs in^9,15,27^, and^29^ operate under linear polarisation, making them inherently susceptible to polarisation mismatch in dynamic body-centric environments where antenna orientation changes continuously; the proposed design overcomes this limitation by generating CP geometry-drivenly without additional complexity. With respect to MIMO diversity, references^9,10,28^, and^29^ lack multi-port capability entirely, while^13^ and^27^ report ECC values of 0.10 and 0.15 respectively — both higher than the proposed ECC of less than 0.05. The isolation of − 12 dB achieved by the proposed design is lower than^14,15,26^, but these designs either operate at non-X-band frequencies, lack CP capability, or do not address wearable SAR compliance; importantly, the ECC below 0.05 confirms that the isolation level does not compromise practical MIMO diversity performance. Finally, on the critical dimension of SAR compliance, only^9,26,27^, and^29^ report SAR values, but none of these simultaneously achieve X-band operation, circular polarisation, and dual-port MIMO in a sub-50 mm footprint. The proposed antenna uniquely combines all five attributes — ultra-compact X-band operation, stable circular polarisation, dual-port MIMO diversity, competitive gain, and verified SAR compliance of 0.96 W/kg (1 g) and 0.726 W/kg (10 g) — within a single passive single-layer architecture, establishing a clear advance over all prior works in the comparison.

## Conclusion

This work presents a compact dual-port circularly polarised MIMO antenna designed for X-band wearable communication applications operating over 9.0–9.3 GHz. The antenna achieves a highly miniaturised footprint of 40 × 15 × 1.6 mm^3^ on an FR-4 substrate, representing a substantial size reduction compared to previously reported X-band CP MIMO designs. Circular polarisation is generated through a geometry-driven feed offset and patch curvature mechanism that excites two orthogonal TM₁₁ degenerate modes with a 90° phase difference, eliminating the need for external matching networks, slot perturbations, or multi-feed excitation structures. The optimised coaxial inset feed depth of 2.505 mm achieves direct 50 Ω impedance matching, confirmed by reflection coefficients below − 15 dB and port isolation better than − 12 dB across the operating band. Stable circular polarisation is maintained throughout the operating bandwidth with axial ratio values below 3 dB. Comprehensive MIMO diversity evaluation confirms envelope correlation coefficients below 0.05, diversity gain approaching 10 dB, channel capacity loss below 0.35 bps/Hz, and balanced mean effective gain of approximately − 3 dB at both ports. On-body SAR analysis using a realistic three-layer hand phantom confirms peak values of 0.96 W/kg for 1 g tissue and 0.726 W/kg for 10 g tissue averaging, remaining well within FCC and ICNIRP regulatory limits respectively. The strong agreement between simulated and measured results validates the reliability of the proposed design for wearable X-band body-centric communication, portable radar sensing, and compact satellite wireless platforms.

## Data Availability

The data used to support the findings of this study are included in the article.

## Declarations

### **Declaration of AI and AI-Assisted Technologies in the Writing Process:**

During the preparation of this manuscript, the authors used Grammarly/AI-assisted language tools to improve grammar, spelling, and language clarity. The authors carefully reviewed and edited the output and take full responsibility for the content of the publication.

## References

[1] Gao, S. et al. Antennas for modern small satellites. *IEEE Antennas Propag. Mag.* **51**(4), 40–56 (2009).

[2] Liddle, J. D. et al. Space science with CubeSats and nanosatellites. *Nat. Astron.* **4**, 1026–1030 (2020).

[3] Vourch, C. J. and Drysdale, T. D. Inter-CubeSat communication with V-band bull’s eye antenna. In: *Proc. 8th Eur. Conf. Antennas Propag. (EuCAP)*, The Hague, Netherlands, pp. 3545–3549 (2014).

[4] Liu, S. et al. A survey on CubeSat missions and their antenna designs. *Electronics* **11**(13), 2021 (2022).

[5] Alqwaifly, N. A., Awan, W. A., Alsaab, N., Alsunaydih, F. N. & Alhassoon, K. Array inspired wideband and high gain antenna with enhanced pattern diversity for 5G mm-wave networks. *Sci. Rep.* **15**, 27383. 10.1038/s41598-025-12868-w (2025). 40721797 10.1038/s41598-025-12868-w PMC12304096

[6] Bandi, R. B., Kothapudi, V. K., Pappula, L., Kalimuthu, R. & Gottapu, S. K. A single-layer S/X-band shared aperture antenna with MIMO characteristics at X-band for airborne synthetic aperture radar applications. *Int. J. Antennas Propag.* **2023**, 1384388 (2023).

[7] Mathur, P. & Kumar, G. Dual-frequency microstrip antenna at S and X bands with higher-order mode suppression technique. *IET Microw. Antennas Propag.* **12**(4), 583–587 (2018).

[8] Serup, D. E., Williams, R. J., Zhang, S., & Pedersen, G. F. Shared aperture dual S- and X-band antenna for nano-satellite applications. In: *Proc. 14th Eur. Conf. Antennas Propag. (EuCAP)*, Copenhagen, Denmark, pp. 1–4 (2020).

[9] Sid, A., Zia, M. A. S. & Khan, M. H. A flexible and wearable dual band bio-based antenna for WBAN applications. *Microelectron. J.* **129**, 105131 (2022).

[10] Manepalli, S. A wideband circularly polarized textile-based microstrip antenna for wearable applications, *Prog. Electromagn. Res. (PIER)*, (2023).

[11] Rivera, A and Stewart, A. Study of spacecraft deployables failures. In: *Proc. Eur. Space Mechanisms Tribology Symp.*, Virtual, (2021).

[12] Mathur, P. & Kumar, G. Dual-frequency microstrip antenna at S and X bands with higher-order mode suppression technique. *IET Microwaves Antennas Propag.* **12**(4), 583–587 (2018).

[13] Shailesh, S. et al. Circularly-polarized sixteen-port flexible UWB MIMO antenna for body-centric communications. *Math. Probl. Eng.* **2024**, 8442770 (2024).

[14] Modak, S. et al. Circularly-polarized diversified MIMO antenna design for mmWave on-body applications using characteristic mode theory, *Int. J. Microw. Wireless Technol.*, (2024).

[15] Hasan, M. M., Islam, M. T., et al., Metamaterial-loaded miniaturized extendable MIMO antenna with enhanced isolation for 5G sub-6 GHz, *J. Electronics*, (2024).

[16] Cappelletti, C., Battistini, S. & Malphrus, B. *CubeSat Handbook: From Mission Design to Operations* (Academic Press, 2020).

[17] Chen, W.-S., Wu, C.-K. & Wong, K.-L. Square-ring microstrip antenna with a cross strip for compact circular polarization operation. *IEEE Trans. Antennas Propag.* **47**(10), 1566–1568 (1999).

[18] Orr, R., Goussetis, G. & Fusco, V. Design method for circularly polarized Fabry-Pérot cavity antennas. *IEEE Trans. Antennas Propag.* **62**(1), 19–26 (2013).

[19] Hossain, M.M. et al. A wideband stacked patch antenna with hybrid perturbations for circular polarization. In: *Proc. IEEE Int. Symp. Antennas Propag. USNC-URSI*, Singapore, pp. 55–56 (2021).

[20] Chahat, N., Sauder, J., Thomson, M., Hodges, R., & Rahmat-Samii, Y. CubeSat deployable Ka-band reflector antenna for deep space missions, In: *Proc. IEEE Int. Symp. Antennas Propag.*, Vancouver, BC, Canada, pp. 2185–2186 (2015).

[21] Hodges, R. E. et al. ISARA — Integrated solar array and reflectarray CubeSat deployable Ka-band antenna. In: *Proc. IEEE Int. Symp. Antennas Propag.*, Vancouver, BC, Canada, pp. 2141–2142 (2015).

[22] Warren, P. A., Steinbeck, J. W., Minelli, R. J., & Mueller, C. Large deployable S-band antenna for a 6U CubeSat. In: *Proc. 29th AIAA/USU Conf. Small Satellites*, Santa Clara, CA, USA, pp. 1–7 (2015).

[23] Tubbal, F. E., Raad, R. & Chin, K. W. A survey and study of planar antennas for pico-satellites. *IEEE Access* **3**, 2590–2612 (2015).

[24] Wang, S., Zhang, L., Zhu, K., & Sun, H. Multi-band shared aperture circularly polarized antenna with metal isolation columns. In: *Proc. IEEE MTT-S Int. Wireless Symp. (IWS)*, Harbin, China, pp. 1–3 (2022).

[25] Xie, M., Wang, Z., Sun, L., Zhu, S., Deng, G., & Cao, Z. An L/S multi-band shared-aperture circularly polarized antenna. In: *Proc. 13th Int. Symp. Antennas, Propag. EM Theory (ISAPE)*, Zhuhai, China, pp. 1–3 (2021).

[26] Kangeyan, R. Circularly polarized implantable MIMO antenna for skin, brain and heart phantoms, *Digital Appl. Commun.*, (2024).

[27] Harlan, L., et al. Reconfigurable ultra-miniaturized MIMO antenna for tissue-independent communication in injectable medical implants, ResearchGate preprint, (2024).

[28] Bhaldar, H. K. Design of circularly polarized compact size wearable antenna, *Int. J. Electron. Commun. (IETE)*, (2023).

[29] Sharma, S. Wearable antenna with reduced SAR using novel FSS reflector, *Prog. Electromagn. Res. (PIER)*, (2023).

[30] Rizvi, S. N. R. et al. A closely spaced two-port MIMO antenna with a radiation null for out-of-band suppressions for 5G Sub-6 GHz applications. *PLoS ONE* **19**(7), e0306446 (2024). 39058682 10.1371/journal.pone.0306446 PMC11280533

